# Precursor-Directed Thermal Synthesis of Copper Catalysts for Tunable CO_2_ to CH_4_ and C_2_H_4_ Conversion at Industrial Current Densities

**DOI:** 10.3390/nano16060386

**Published:** 2026-03-23

**Authors:** Hunter B. Vibbert, Luqman Azhari, Nathan Rafisiman, Emma Olson, Bing Tan, Nicholas G. Pavlopoulos

**Affiliations:** 1 Johns Hopkins Applied Physics Laboratory, Research and Exploratory Development Department, 11100 Johns Hopkins Rd, Laurel, MD 20723, USA; 2 Pacific Industrial Development Corporation, 4788 Runway Blvd, Ann Arbor, MI 48108, USA

**Keywords:** catalysts, electrochemistry, CO_2_ reduction, precursor-directed, scalable CO_2_-reduction, CO_2_-to-fuel, CO_2_-to-methane, CO_2_-to-ethylene

## Abstract

Scalable copper catalysts for electrochemical CO_2_ reduction have been prepared through precursor-directed thermal synthesis, enabling tunable conversion to CH_4_ and C_2_H_4_ at industrial current densities. Thermal treatment of distinct copper precursor salts was found to yield nanostructured catalysts with composition- and morphology-dependent selectivity, and high Faradaic efficiencies under flow conditions. This simple, low-cost process demonstrates that precursor chemistry can control active phase formation and product distribution, providing a practical route toward scalable CO_2_ electroreduction.

## 1. Introduction

The development of scalable, cost-effective catalysts for electrochemical CO_2_ reduction processes still faces major technical hurdles to compete with existing fuel production technologies. More than ever, developing these processes is important to decrease reliance on fossil fuels and develop new industries that can rely on circular carbon and hydrogen economies [1,2,3]. Electrochemical CO_2_ reduction is promising in this regard [4,5], as the technique holds the potential to create a multitude of industrially relevant products with cost-effective potential [6]. In most electrochemical CO_2_ reduction approaches, electrocatalysts, often copper-based [7,8,9,10], are employed to catalytically reduce CO_2_. Depending on the composition and morphology of the catalyst, a diversity of gaseous- and liquid-phase products can result, some with high selectivity and current densities [7,9]. In principle, these products could be used to supplement or replace conventional fossil fuel-derived materials, providing an entry into a circular carbon economy [11]. Among value-added CO_2_ reduction reaction gaseous products are methane and ethylene [12,13], which can be easily integrated into current industrial processes. Ethylene, for instance, is a widely used commodity chemically produced via steam cracking. CO_2_ reduction reaction processes, if properly scaled and executed, could potentially be used as surrogate materials for the approach. However, at present, nearly all catalyst materials are produced in ways that lack the potential for scaling, industrial integration, or optimization.

The most common family of CO_2_ reduction catalysts towards C1 and C2 hydrocarbons contain copper [14,15,16,17,18]. While conceptually simple, a vast array of these catalyst materials have been developed to provide precise control for selectivity towards specific reduction products through tuning of parameters such as particle size, morphology, and crystal facet distribution. Various methods of synthesis exist for these catalytic materials, which commonly include direct electrochemical deposition on electrodes [19,20], synthesis of nano-scaled catalyst materials [21,22], and the deposition of inks on carbon substrates [23]. Although these electro-synthesized working electrode materials can yield a wide range of carbon-containing products, challenges remain regarding their scalability and reproducibility. In contrast, ink-based preparation methods, when paired with readily available and scalable catalyst formulations, offer a more practical route toward large-scale production using existing technologies and infrastructure.

Here, we report the development of a family of widely scalable, tunable systems utilizing catalysts prepared by simple thermal treatment (250 °C in air or N_2_ atmosphere) of low-cost copper precursors (**1** = CuCO_3_; **2** = Cu(acac)_2_; **3** = Cu(OH)_2_). Depending on the precursor and heat treatment, we find that, in a flow cell configuration, the catalysts exhibit selectivity towards CH_4_ or H_2_C=CH_2_ products with high current densities (250 mA cm^−2^) and total Faradaic efficiencies. Despite the relative simplicity of the thermal annealing approach, industrially relevant CO_2_ electroreduction systems require catalysts that can be produced at the kilogram scale, are integrated into high-throughput fabrication methods, and that deliver robust performance under commercially viable conditions. As a result, we focused on developing a straightforward process for producing nanostructured copper catalysts supported on carbon scaffold that can be scaled to the kilogram level at <$10/kg [4], achieving high current densities (250 mA cm^−2^) with appreciable selectivity (up to 30% FE) for ethylene, and maintaining a level of C1–C2 carbon conversion products that rivals more complex [24,25,26] catalyst architectures on a total molar basis while offering a clear path to manufacturing integration.

## 2. Materials and Methods

**General Procedures.** Manipulations were performed in a chemical fume hood, in air, or on a standard laboratory benchtop with standard techniques. Reagents used for preparation were procured from commercial sources and used as received. Pyrolysis synthesis was conducted with an MTI tube furnace (MTI, Richmond, CA, USA) or Thermo Scientific muffle furnace (Thermo Scientific, Waltham, MA, USA). ^1^H NMR was recorded at room temperature on a Bruker Avance 400 spectrometer. Chemical shifts were measured relative to internal solvent resonances [27]. Scanning Electron Microscopy was measured using a Carl Zeiss EVO MA10 SEM (Zeiss, Oberkochen, Germany) or a Thermo Scientific Helios G4 UC FIBSEM. Powder X-Ray Diffraction Studies were recorded on a Rigaku Miniflex II (Rigaku, Tokyo, Japan). Gas Analysis via flow FT-IR was recorded on a MKS Multigas 2030 HS (MKS Instruments, Andover, MA, USA). Carbon content was determined using Eltra ELEMENTRAC CS-I (ELTRA, Haan, Germany).

**Catalyst Preparation.** CuCO_3_ (99%, Noah Chemicals), Cu(OH)_2_ (Noah Chemicals), and copper acetylacetonate (98%, Beantown Chemicals) were used as catalyst precursors. Powders were used as-is and subjected to heat treatment. Heat treatment was performed at 250 °C (4 h treatment) either in air or under a N_2_ atmosphere.

**Working Electrode Preparation.** The working electrode was constructed utilizing a gas diffusion electrode (GDE)-supported catalyst. These were fabricated by preparing a catalyst ink comprising catalyst (0.005 g), Sustainion XA-9 Alkaline Ionomer (25 μL, 5% solutions obtained from Dioxide Materials), and ethanol (0.5 mL). The ink was ultrasonicated (240 W at 40 kHz at room temperature, 0.5 h) and dropcast onto the microporous side of a 24 mm GDL disk (Freudenberg H_23_C_6_, (FuelCell Store, College Station, TX, USA)). The disk was heated at 90 °C for at least 15 min to evaporate all solvent. Targets for solids loadings were 0.8 mg cm^−2^.

**Electrochemical Procedures.** Tests were run with a Gamry 1010 potentiostat (Gamry Instruments, Warminster, PA, USA). CO_2_ reduction reactions were conducted in an electrochemical flow cell (DekResearch) constructed of PEEK. A three-electrode configuration was employed consisting of a Ag/AgCl (1 KCl) reference electrode and a Ni foam counter. A Fumasep PK-130 anion exchange membrane, soaked in an aqueous solution of KOH (1 M), separated the catholyte and anolyte chambers. The working electrode area was maintained at 1 cm^2^ using a silicone gasket. During operation, the CO_2_ flow rate was at 200 sccm. A KOH solution (1 M) was used in the catholyte and anolyte. Anolyte and catholyte flow were set with a peristaltic pump (Masterflex L/S (Avantor, Radnor, PA, USA), 4.25 mL min^−1^) in reservoirs (20 mL). Electrodes were conditioned prior to electrolysis with linear sweep voltammetry (2 sweeps, −0.2 V–2.0 V, v Ag/AgCl). Linear sweep voltammetry was conducted in the presence and absence of CO_2_ (Airgas, Radnor, PA, USA) stream (200 sccm) using a Gamry 1010 potentiostat with a standard 3-electrode configuration consisting of a Ag/AgCl (1 KCl) reference, fabricated working electrode, and Ni foam counter electrode. Onset potentials were approximated from the 5% intensity difference in the LSV curve. Sweeps were recorded from −0.2 V–2.0 V, v Ag/AgCl. Example voltammograms are shown in Figures S13–S18.

**Electrochemical Catalysis Procedures.** Tests were run with a Gamry 1010 potentiostat. CO_2_ reduction reactions were conducted in an electrochemical flow cell (DEK Research, Hong Kong, China) constructed of PEEK. A three-electrode configuration was employed consisting of a Ag/AgCl (1 KCl) reference electrode and a Ni foam counter. A Fumasep PK-130 anion exchange membrane, soaked in an aqueous solution of KOH (1 M), separated the catholyte and anolyte chambers. The working electrode area was maintained at 1 cm^2^ using a silicone gasket. During operation, CO_2_ flow rate was at 200 sccm. A KOH solution (1 M) was used in the catholyte and anolyte. Anolyte and catholyte flow were set with a peristaltic pump in 20 mL reservoirs. Electrodes were conditioned prior to electrolysis with linear sweep voltammetry (2 sweeps, −0.2 V–2.0 V, v Ag/AgCl). Tests were conducted with a current density of 0.25 A cm^2^. Gas evolution was monitored by an in-line FT-IR and liquid phase products were monitored by NMR spectroscopy.

## 3. Results and Discussion

In contrast to other precisely defined catalyst systems, **1–3** are easily generated from as-received commercial powders or by thermal treatment at low temperatures (250 °C, see Supporting Information for details). Samples **1–3** span a range of catalyst precursors containing inorganic carbon, organic carbon, and minimal carbon content (e.g., CO_3_, acac, and OH, Table S1) and exhibit different crystalline structures as evidenced by SEM, TEM (Figures S10 and S11), and p-XRD analysis (Figure S12). SEM images show distinct features representative of the untreated samples **1–3.** Across the viewing window of the images, samples **1–3** contain heterogeneous features, presumably due to differences in preparation steps (Figures S1–S9). In magnified regions, sample **1-untreated** exhibits packed needle-like primary particle structures that form tight micrometer-scale spherical aggregates (~5–10 µm). In contrast, sample **2-untreated**, which contains an organic counter-ion source, acetylacetone, appears to manifest with fewer aggregates composed of larger, micron-sized plate-like primary particles. Lastly, sample **3-untreated** is composed of loose agglomerations of nanosized, needle-like primary particles.

Upon heat treatment, smooth conversion from the starting structures to new crystalline phases are observed. For sample **1**, new features that are commensurate with the formation of a CuO phase [28] result from heat treatment in both air and N_2_. These structural features are in line with expected calcination products resulting from thermal decomposition of CuCO_3_. Treatment under air or N_2_ does not appear to provide any differences in secondary phases. However, thermal treatment under N_2_ results in larger crystallite sizes, as evidenced by the narrower full-width half-maximums compared to the air-treated counterpart. Copper(II) oxide, again, appears to be the predominant product upon heat treatment of both samples **2** and **3** in air. Under N_2_, however, product distribution is more complex. For sample **2-N_2_**, the formation of multiple new crystalline phases are observed, which can be indexed to the patterns of a mixed phase of both Cu_2_O and reduced Cu(0) [29,30]. Presumably, this results in the partial or complete reduction of the copper center upon the decomposition of the acetylacetone ligand [31]. Small quantities of Cu_2_O are observable in sample **2-air** as well. CuO is the predominant crystal phase which implies that, while O_2_ is present, decomposition of the organic ligand still leads to partial reduction of the copper ion. The **3-N_2_** sample is also found to contain a mixture of both CuO and Cu_2_O phases, with significant fractions of each phase present in the sample.

**Figure 1:**
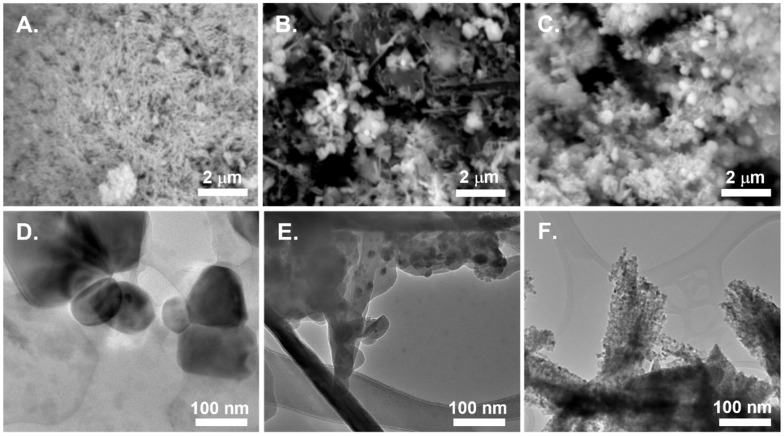
SEM and TEM images of nitrogen-treated powders of (**A**,**D**) **1**-N_2_; (**B**,**E**) **2**-N_2_; and (**C**,**F**) **3**-N_2_.

SEM images of the treated powders (Figure 1) show several changes in primary particle size and shape after heating. The structural features of the **1-N_2_** sample still appear as spherical aggregates of small, needle-like primary particles. However, the microstructures of samples **2** and **3**, when treated, are significantly altered. For instance, sample **2-N_2_** powders are composed of small quasi-spherical copper oxide particles, generated from thermal treatment of the organic ligand. In contrast, sample **2-air** (Figure S8) is composed of quasi-spherical agglomerates containing copper oxide. For sample **3-N_2_**, a mixture of dense copper spheres embedded in a mixture of smaller primary particles is observed. Interestingly, sample **3-air** has a nearly unchanged morphology compared to its untreated counterpart. The differences in morphology, along with the initial crystal phase, are expected to impact selectivity of the catalyst during CO_2_ reduction. Under TEM imaging (Figure 1), all samples appear to be composed of copper/copper oxide nanoclusters embedded in amorphous carbon coatings (or carbon flakes in the case of sample **2-N_2_**). Sample **3-air**, however, is composed of clusters of distinct rods, which themselves are composed of fine CuO nanoparticles (Figure S11).

**Figure 2:**
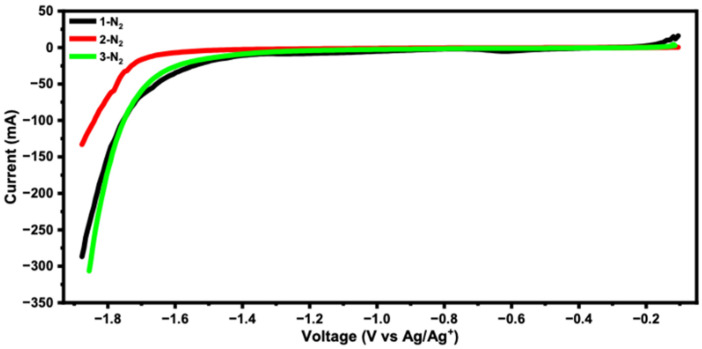
LSV of catalysts prepared under nitrogen annealing under CO_2_ atmosphere, highlighting high currents and onset potentials between −1.4 and −1.75 V (V vs. Ag/AgCl).

Electrochemical characterization of the catalyst inks, prepared via formulation of the synthesized powders with carbon black and ethanol (see SI for synthetic details), was performed to understand how structural similarities and differences could give rise to different onset potentials for catalysis. All LSV data are set forth in the SI (Figures S13–S18), with key data summarized in Figure 2. In general, the catalytic onset features of these fabricated electrodes from catalyst inks show a wide range of potentials for CO_2_ reduction. These features span a range between −1.4 and −1.75 V (V v. Ag/AgCl). At the most negative end of this range are the catalysts containing untreated CuCO_3_ and metallic copper particles, samples **1**-untreated and **2**-N_2_. All catalysts demonstrated high activity under conditions of electrolysis and were tested for product distributions in an electrochemical flow cell configuration. The differences in onset potential indicate a difference in selectivity of CO_2_ reduction products, as products such as CH_4_ are typically generated at more negative potentials than products such as C_2_H_4_ and CO.

**Figure 3:**
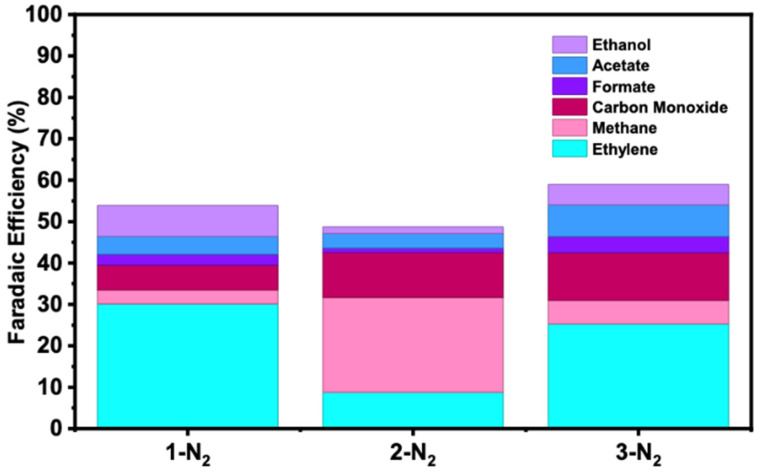
Faradaic efficiencies of gaseous and liquid carbon-containing products for samples **1–3** with nitrogen treatment over the course of an 1800 s constant current. (250 mA/cm^2^). Gaseous products were quantified by FT-IR and liquid products were quantified via ^1^H NMR with dimethylsulfone as an internal standard.

Flow electrochemistry of these materials was accomplished via reaction screening, with gaseous products monitored via in-line connection to an FT-IR analyzer. Under constant current electrolysis (250 mA/cm^2^), several gaseous hydrocarbon products were observed, with experiments initially monitored during the course of the 1800 s operation. Faradaic efficiency data are shown for nitrogen-treated samples in Figure 3. Other Faradaic efficiency data is reported in the Supporting Information. Under this high current density load, both methane and ethylene were produced in appreciable quantities, particularly for nitrogen-treated samples. For instance, sample **1-N_2_** exhibited the highest Faradaic efficiency for ethylene (30.09%, Figure 3)—an impressive result given the simplicity of the designed catalyst structures. It was found that variations of samples **1–3** were reasonably selective for ethylene or methane as well. For instance, nitrogen-treated **1**-N_2_ and air-treated **3**-**air** were found to be the most selective for ethylene (8.9:1 and 15.7:1 ratio, respectively, Tables S5 and S6). Further, these catalysts also exhibit good selectivity factors against CO (5.0:1 and 2.9:1 respectively). In contrast, sample **1**-untreated is the most selective catalyst for methane, with a 17:4:1 ratio against ethylene and a 11.4:1 ratio against CO.

In addition to the gaseous products evaluated using FT-IR, liquid products were evaluated using NMR spectroscopy [32] to understand the variation and relative quantities produced (Figures S29–S37). Total quantities of liquid products were evaluated in both the catholyte and anolyte chambers and added together, since the membrane separator allowed free passage of anionic molecules (e.g., formate and acetate) that could be reasonably found in the product of these reactions. A general method for quantifying liquid products is set forth in the Supporting Information (Tables S2–S6). In general, the total Faradaic efficiencies of the liquid products are such that formate ~ acetate > ethanol. However, slightly larger efficiencies for ethanol production are found for samples **1-N_2_** (7.41%) and **2**-**air** (6.44%). Product selectivity was heavily dependent on the copper precursor used and the heat treatment applied (Figure 1). Sample **3** was ethylene-selective in all conditions tested, which may be attributed to the flake-like morphology of the initial copper oxide/hydroxide, which was unaffected from either heat treatment in air or N_2_. Treatment under N_2_ conditions for all precursor types was found to improve the overall faradaic efficiency towards CO_2_ reduction versus the untreated or air-treated samples. The improvement in CO_2_ reduction efficiency may be attributed to partial nitridation-oxidation of the copper oxide or metallic copper. Such a mechanism has been observed to result in the increased generation of grain boundaries, which may have contributed to an increase in the overall catalytic activity of the sample [33]. In the majority of cases, the lifetimes of samples **1–3** under the conditions employed in these experiments were less than 1800 s (Figures S19–S27). Upon disassembling the flow reactor, a large quantity of colorless buildup was observed on the gas diffusion electrode, presumably K_2_CO_3_ formation from the interface between CO_2_ and the KOH electrolyte solution. This residue facilitates the formation and precipitation of carbonate salts which cause failure in the system [34]. Interestingly, upon washing the electrode with DI water, nearly full electrocatalytic activity was recovered (Figure S28), suggesting that the main source of the rapid activity loss observed was due to flooding of the gas diffusion layer and subsequent mass transfer limitations of CO_2_ to the catalyst surface, and not necessarily due to catalyst over-reduction or degradation at these timescales, as previously reported [35] for other catalyst systems.

## 4. Conclusions

Collectively, this work has demonstrated that low-cost, potentially scalable electrocatalysts could be utilized at high current densities (250 mA/cm^2^) to reduce CO_2_ to value-added products. Depending on the catalyst treatment and original composition, selective electrocatalysts for ethylene and methane could be developed. These catalysts show high activity, possess useful lifetimes, and can be synthesized in bulk quantities. Further, owing to their relative simplicity, ease of preparation, and spray coating compatibility, these materials may be incorporated along with leading strategies for limiting electrode fouling, suppressing the hydrogen evolution reaction, and tuning product distribution to enable low cost and highly selective CO_2_ reduction catalysts. Taken together, we envision that this work marks the initial steps of the rapid development of low-cost electrocatalytic materials to produce useful fuels.

## Data Availability

Data is contained within the article or Supplementary Materials.

## Supplementary Materials

The following supporting information can be downloaded at: https://www.mdpi.com/article/10.3390/nano16060386/s1, Table S1. Carbon content (wt%) of catalysts 1–3 under untreated, N_2^−^_, and air-treated conditions, showing strong precursor- and atmosphere-dependent carbon retention. Figures S1–S3. SEM images of untreated catalysts 1–3 showing baseline morphologies. Figures S4–S6. SEM images of N_2_-treated catalysts 1–3 showing morphology after inert thermal processing. Figures S7–S9. SEM images of air-treated catalysts 1–3 showing morphology after oxidative treatment. Figures S10 and S11. TEM micrographs of air-treated catalysts showing nanoscale particle structure. Figure S12. P-XRD patterns comparing phase composition across precursors and treatment conditions. Figures S13–S15. LSV of catalysts under N_2_ showing background electrochemical behavior. Figures S16–S18. LSV under CO_2_ showing catalytic response differences. Figures S19–S21. Gas product distributions for untreated catalysts showing baseline activity. Figures S22–S24. Gas product distributions for N_2_-treated catalysts. Figures S25–S27. Gas product distributions for air-treated catalysts. Figure S28. Product distribution during catalyst regeneration over time. Table S2. Relative ^1^H NMR integrations for formate, acetate, and ethanol in catholyte and anolyte samples. Table S3. Standardized liquid-product concentrations calculated from the NMR integrations. Table S4. Total quantities of liquid products generated in flow-cell electrolysis. Table S5. Faradaic efficiencies for gaseous and liquid products across catalysts 1–3, showing treatment-dependent selectivity. Table S6. Peak faradaic efficiencies for gaseous products, highlighting differences in maximum CO, CH_4_, and C_2_H_4_ selectivity across catalyst treatments. Figures S29–S31. ^1^H NMR of untreated catalyst solutions showing liquid products. Figures S32–S34. ^1^H NMR of N_2_-treated catalyst solutions. Figures S35–S37. ^1^H NMR of air-treated catalysts solutions. Cost analysis: Estimated material and energy costs for catalyst production, giving a total projected cost of $9.04 kg^−1^ catalyst based on precursor and furnace-energy inputs.
