# Supplementary material for: Precursor-Directed Thermal Synthesis of Copper Catalysts for Tunable CO2 to CH4 and C2H4 Conversion at Industrial Current Densities
Source: Nanomaterials (Basel). 2026 Mar 23;16(6):386. doi: 10.3390/nano16060386 (PMC13029196; doi:10.3390/nano16060386)
Supplement: Supplementary file 1 [file nanomaterials-16-00386-s001.zip › nanomaterials-4147347-supplementary.pdf]

SUPPORTING INFORMATION

Precursor-Directed Thermal Synthesis of Copper Catalysts for Tunable CO_2 to CH_4 and C_2H_4 Conversion at Industrial Current Densities

AUTHORS

*Hunter B. Vibbert,[1¥] Luqman Azhari,[2¥] Nathan Rafisiman,[1] Emma Olson,[1] Bing Tan,[2] Nicholas G Pavlopoulos[1]**

1. Johns Hopkins University Applied Physics Laboratory, 11100 Johns Hopkins Road, Laurel MD 20723
2. Pacific Industrial Development Corporation, 4788 Runway Blvd. Ann Arbor, MI 48108

[¥]These authors contributed equally

E-mail: Nicholas.Pavlopoulos@jhuapl.edu

Contents

1. Synthesis and Characterization of Catalyst Materials 2-12
2. Electrochemical Characterization 12-15
3. Electrochemical Catalysis..15-20
4.Product Analysis 20-35
5.Cost Analysis 36
6.References...37

1. Synthesis and Characterization of Catalyst Materials

Table S1. Carbon Content (wt%) Recorded from Catalysts Preparations.

Precursor	Untreated	N_2	Air
$CuCO_3$ (**1**)	5.74	0.44	0.45
$Cu(acac)_2$ **(2)**	45.48	17.19	1.17
$Cu(OH)_2$ **(3)**	0.23	2.38	0.26

1.4 SEM Images of Catalyst Materials

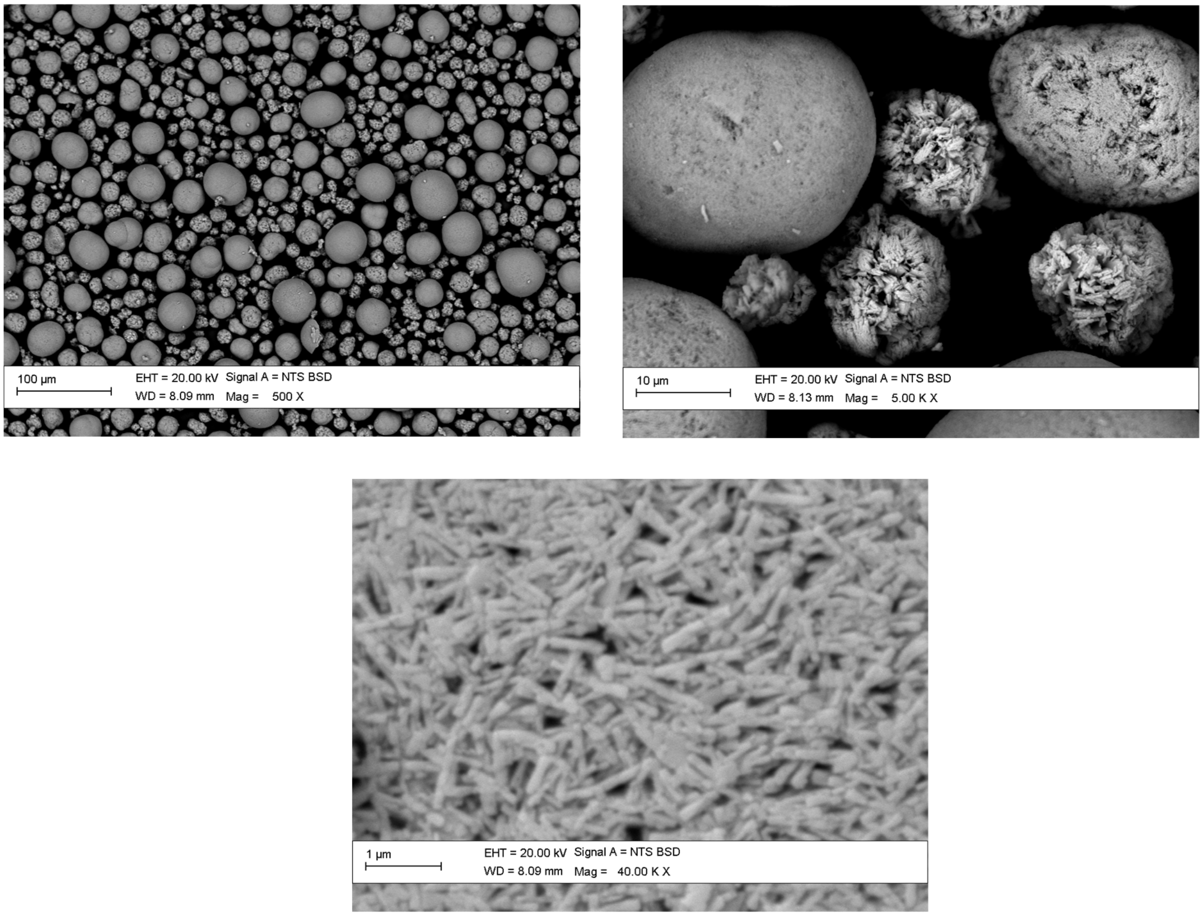

Figure S1. SEM images of **1**-untreated.

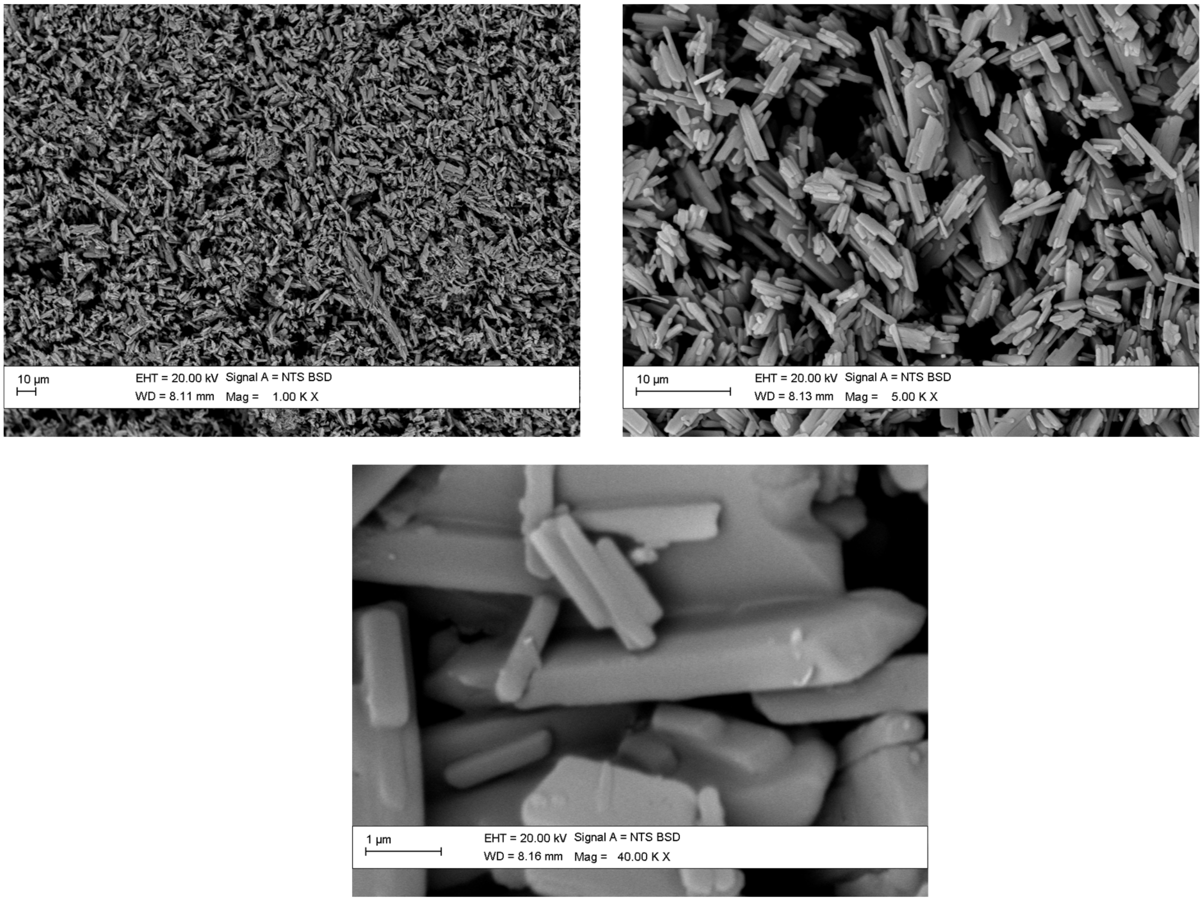

Figure S2. SEM images of **2**-untreated.

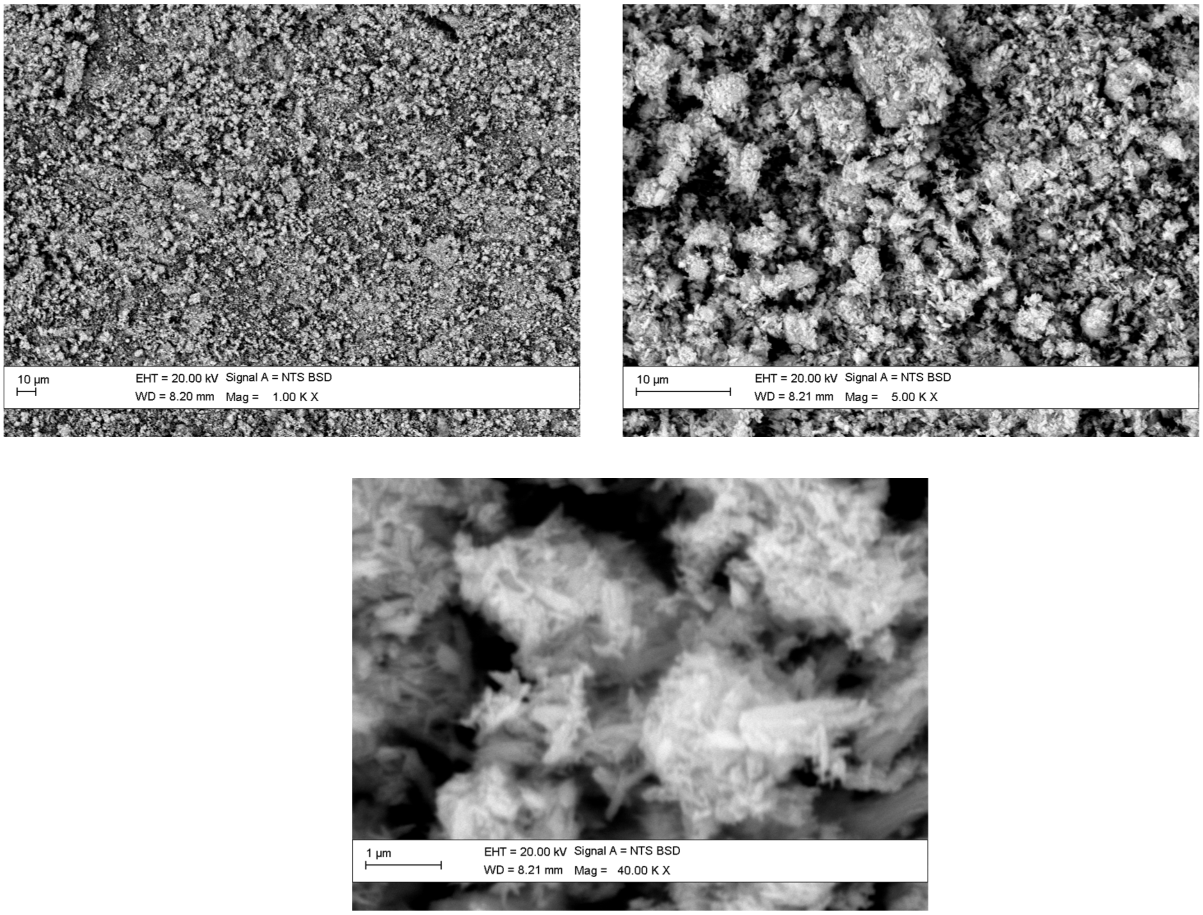

Figure S3. SEM images of **3**-untreated.

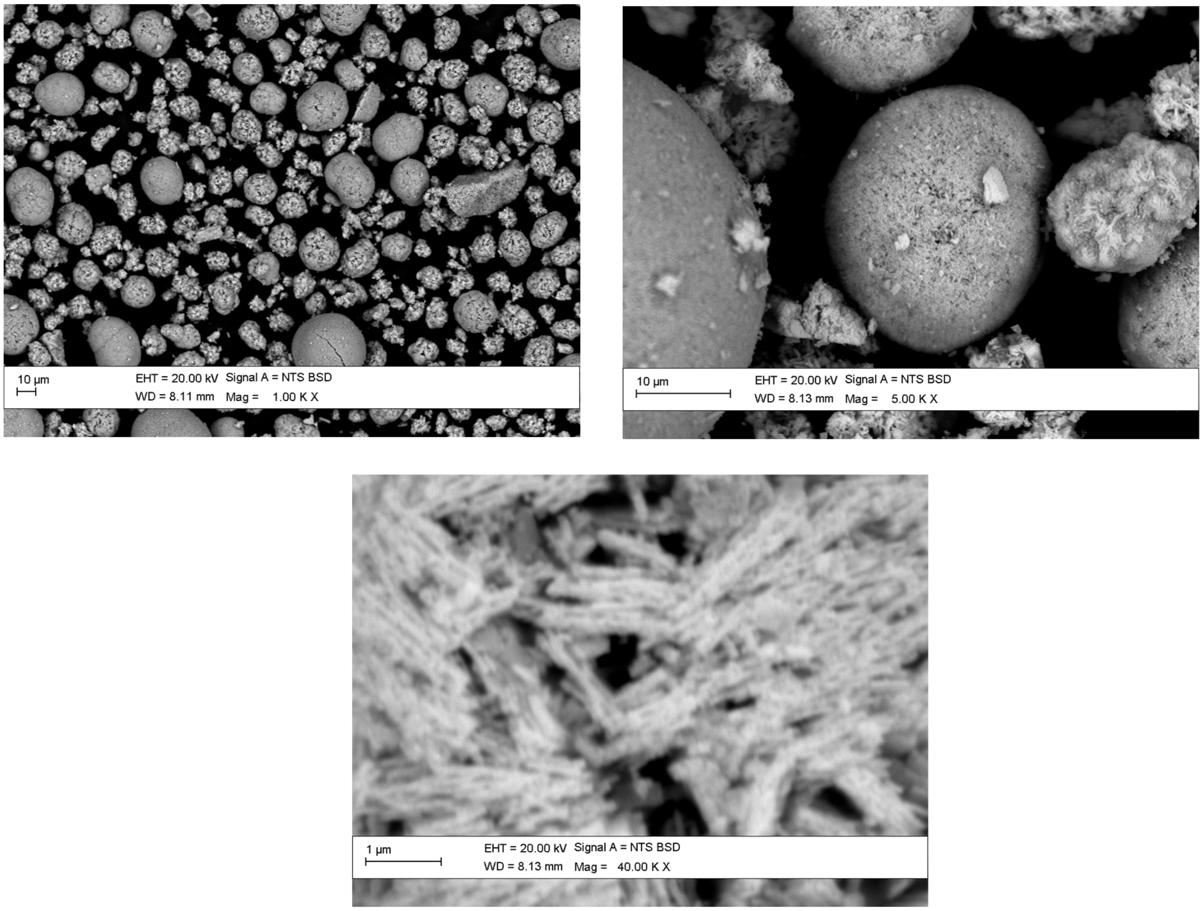

Figure S4. SEM images of **1**-N_2.

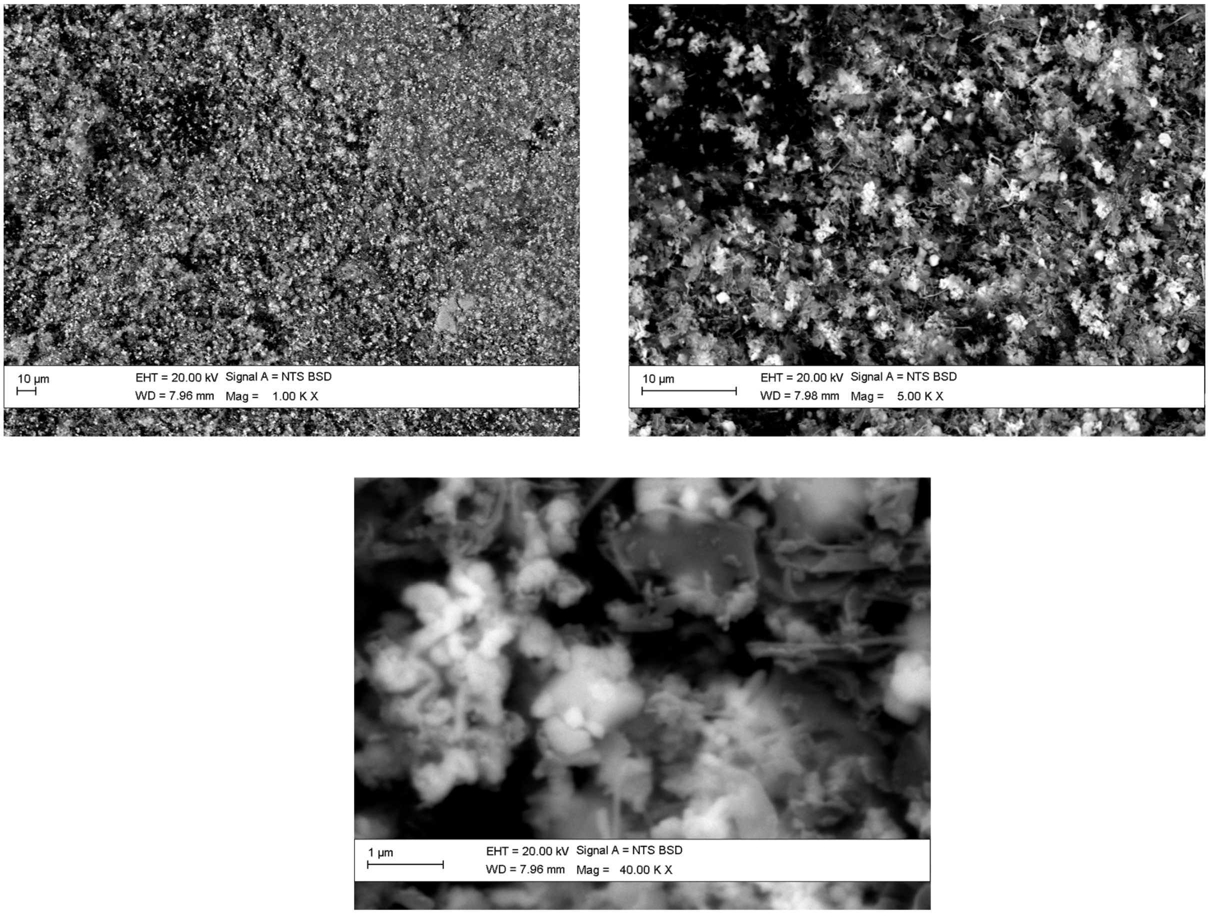

Figure S5. SEM images of **2**-N_2.

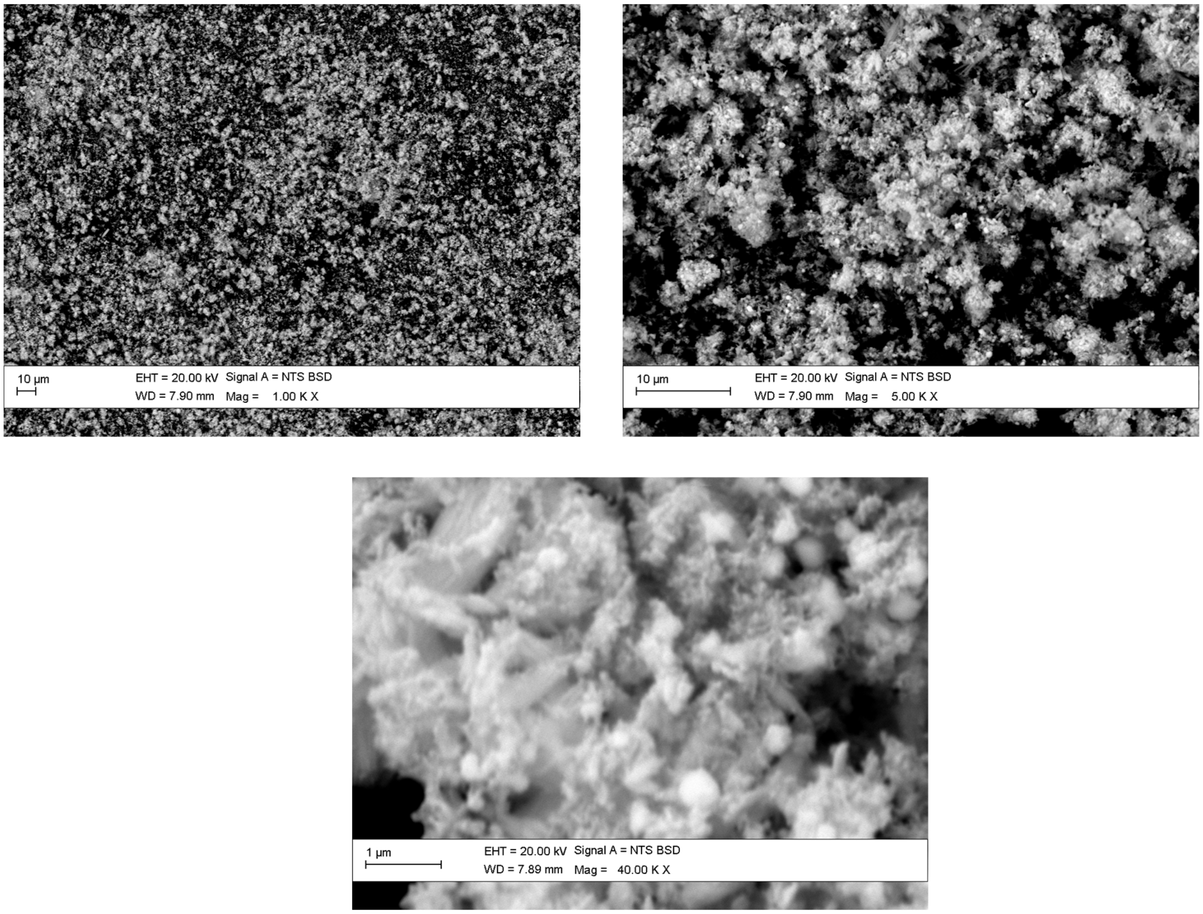

Figure S6. SEM images of **3**-N_2.

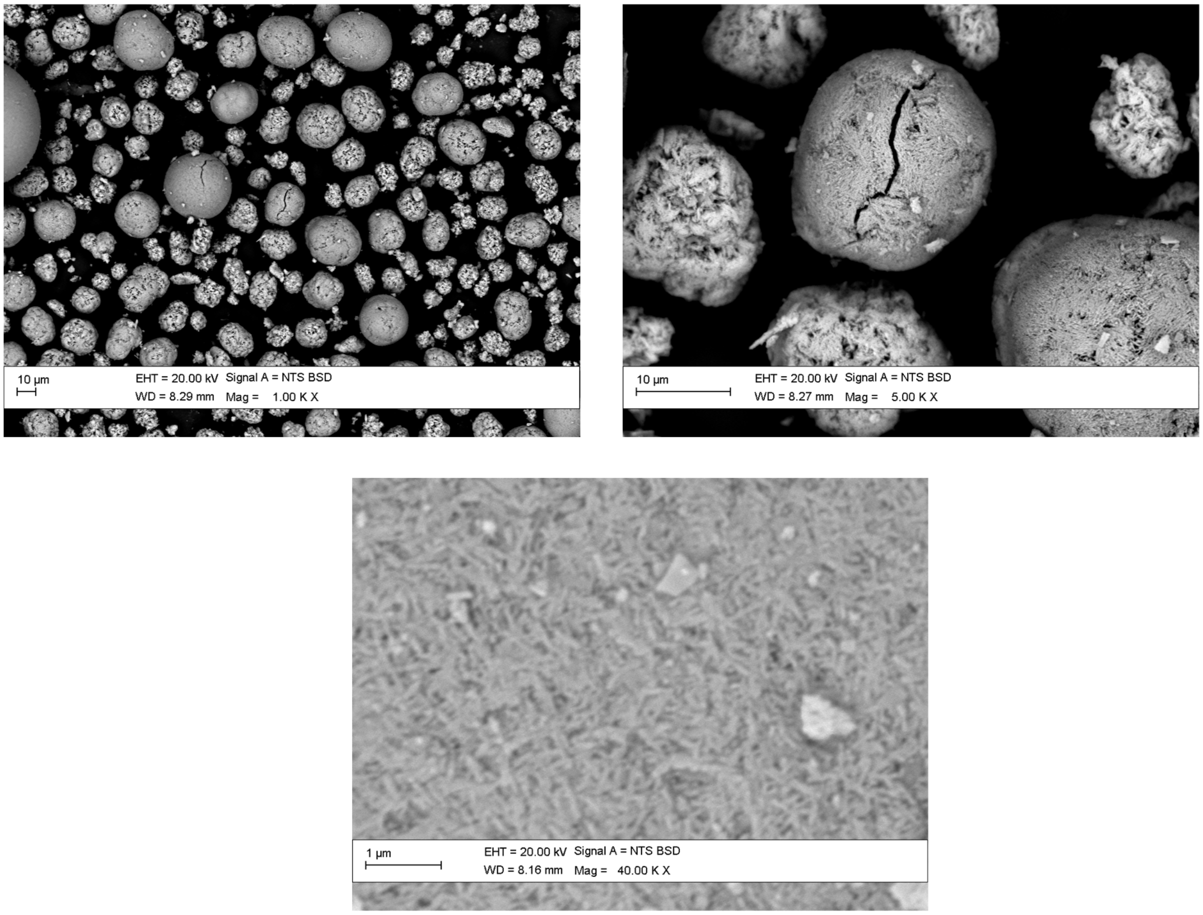

Figure S7. SEM images of **1**-air.

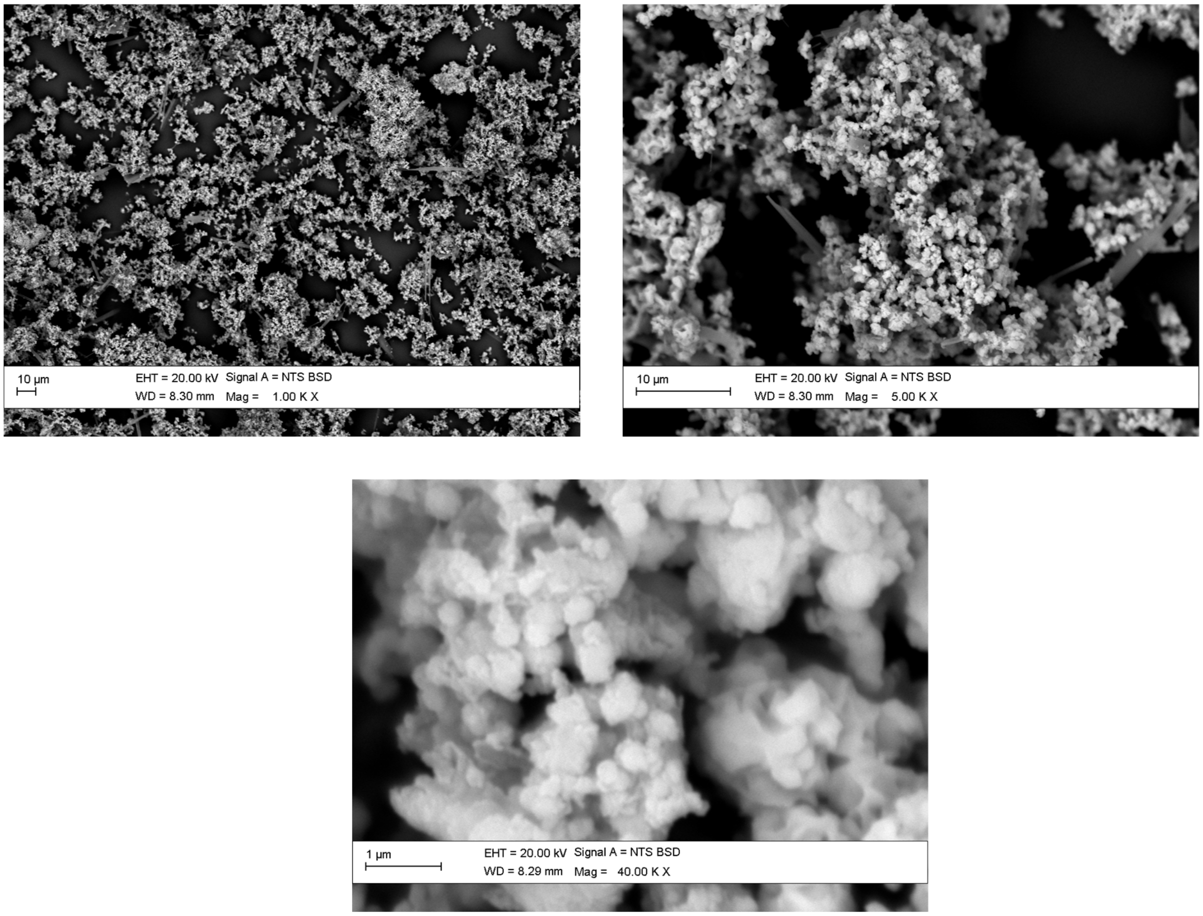

Figure S8. SEM images of **2**-air.

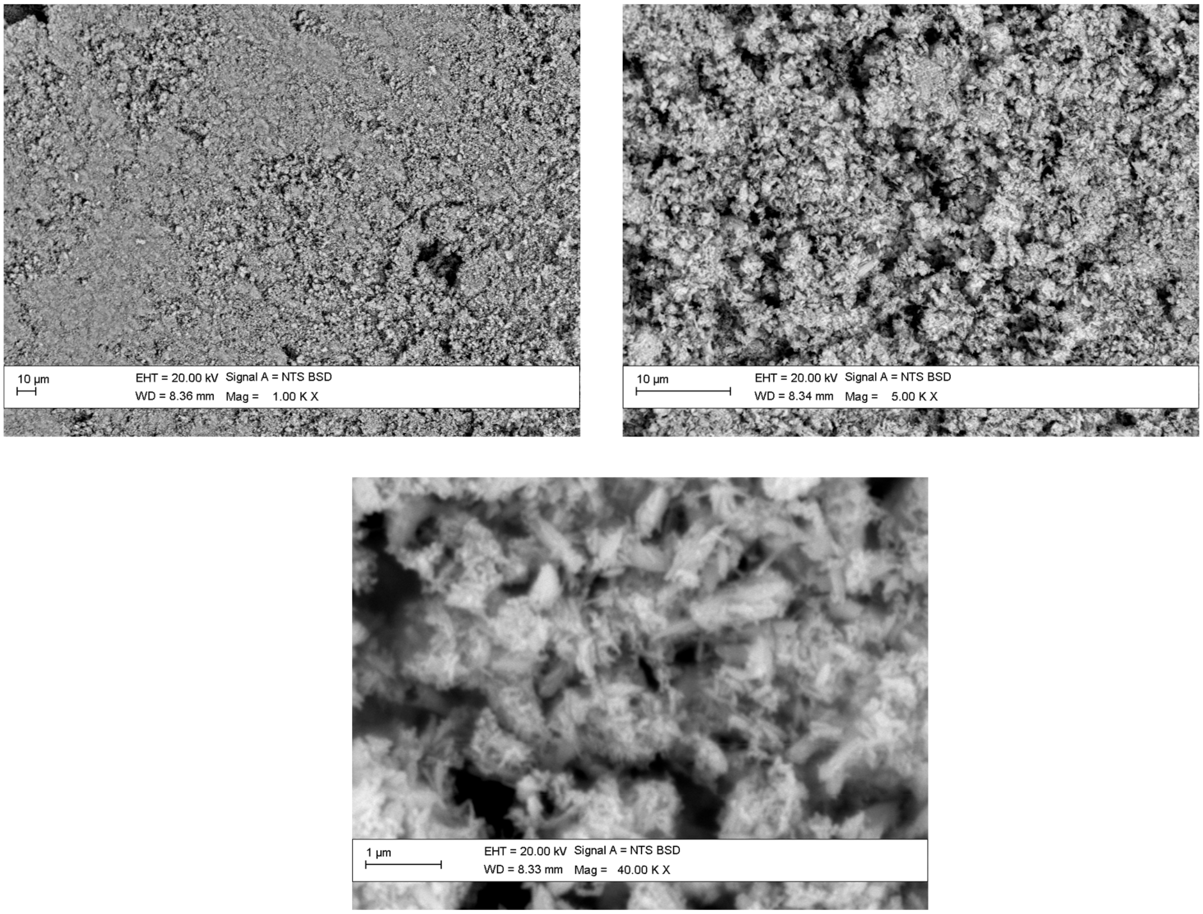

Figure S9. SEM images of **3**-air.

1.5 TEM Images of Catalyst Materials

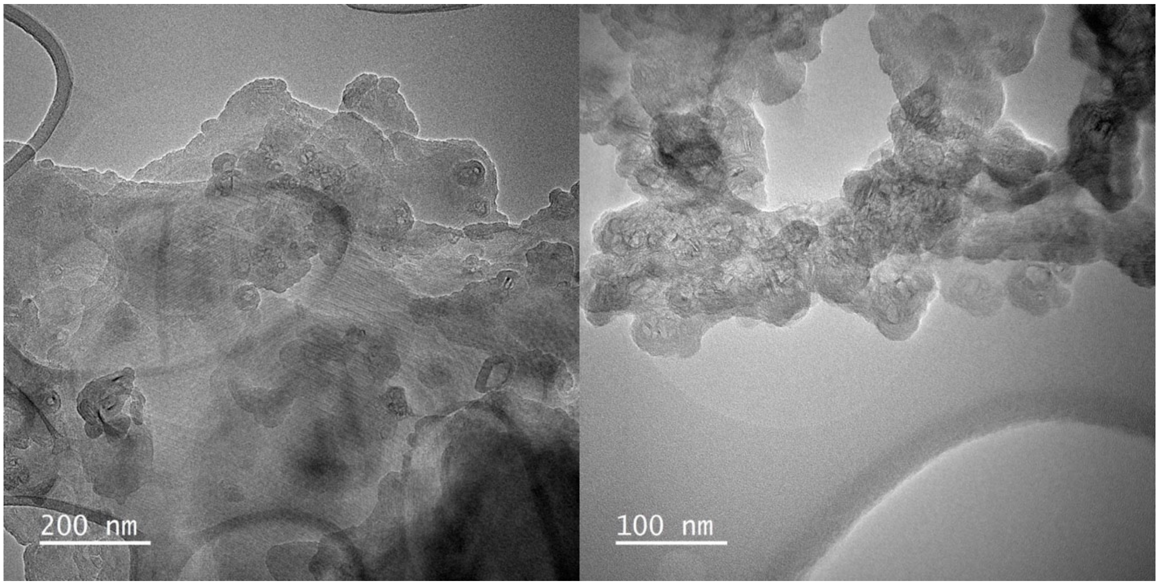

Figure S10. TEM micrographs of **1**-air (left), **2**-air (right),

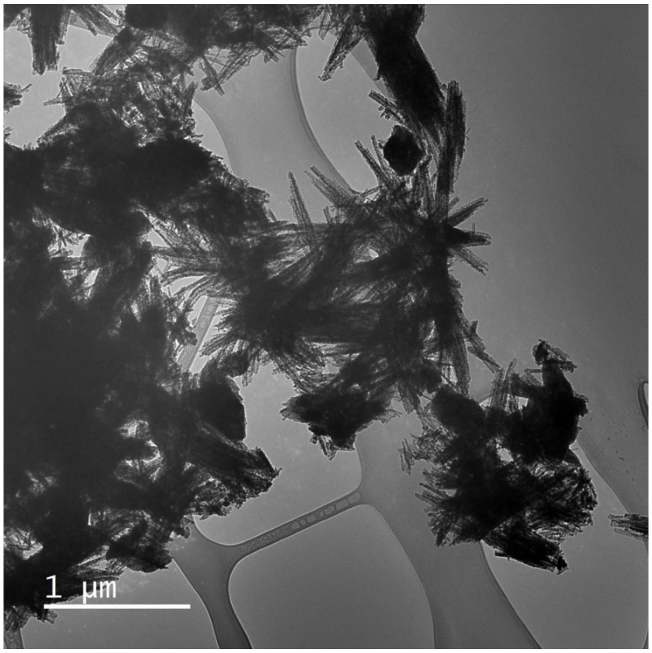

Figure S11. TEM micrograph of **3-**air

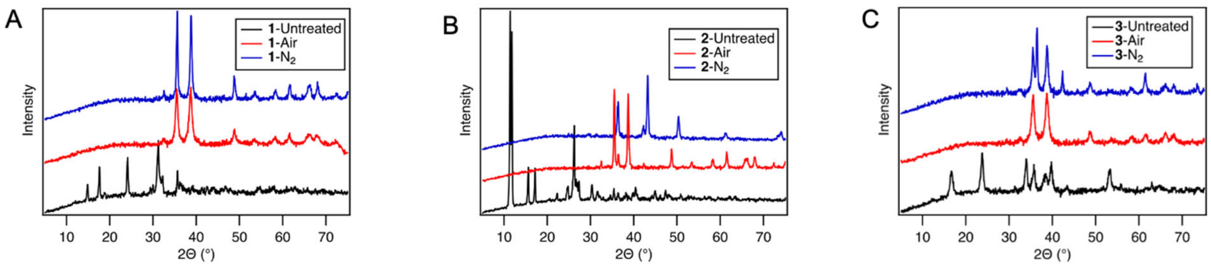

Figure S12. P-XRD data of **1-3** under various treatment conditions.

2. Electrochemical Characterization

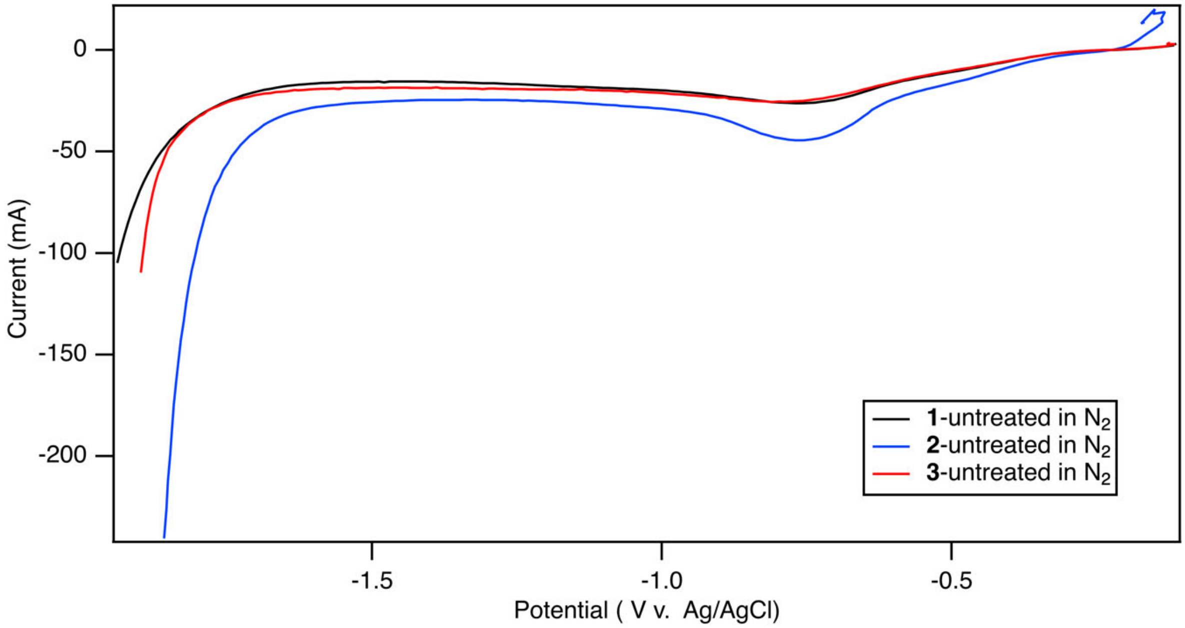

Figure S13. LSV of **1,2,3-**untreated under N_2.

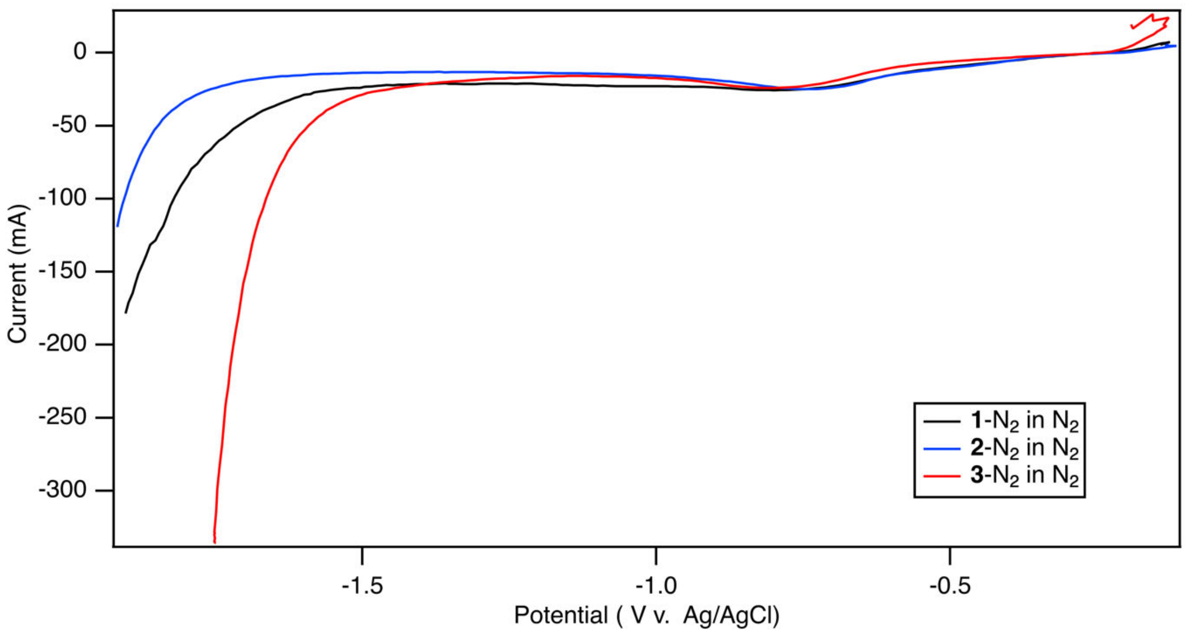

Figure S14. LSV of **1,2,3-**N_2 under N_2.

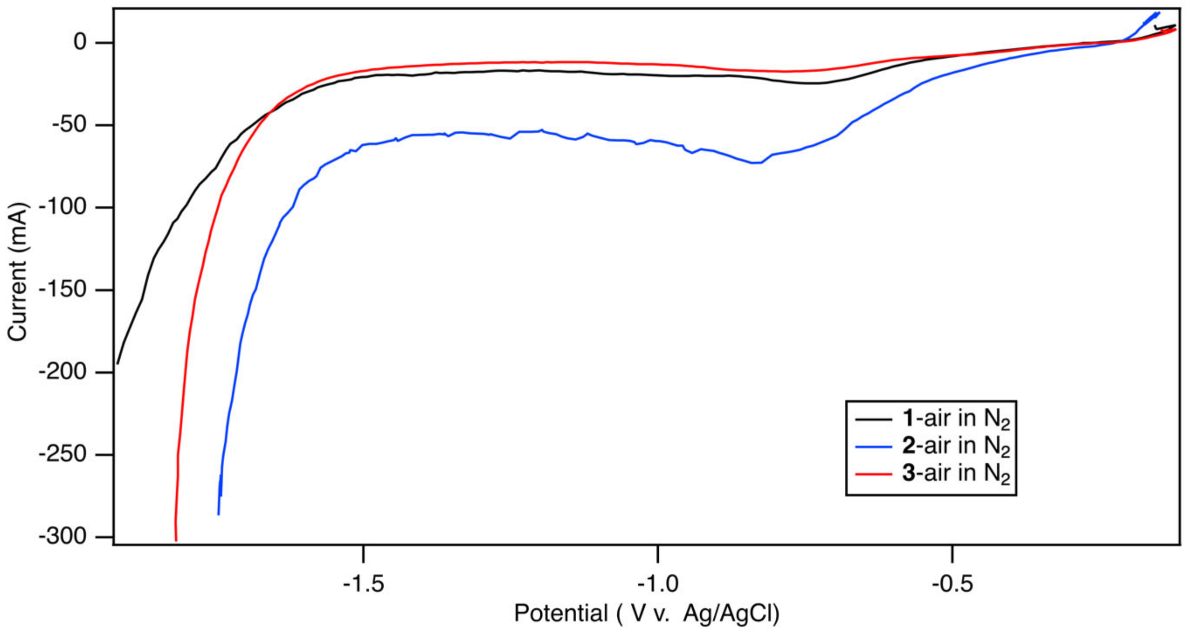

Figure S15. LSV of **1,2,3-**air under N_2.

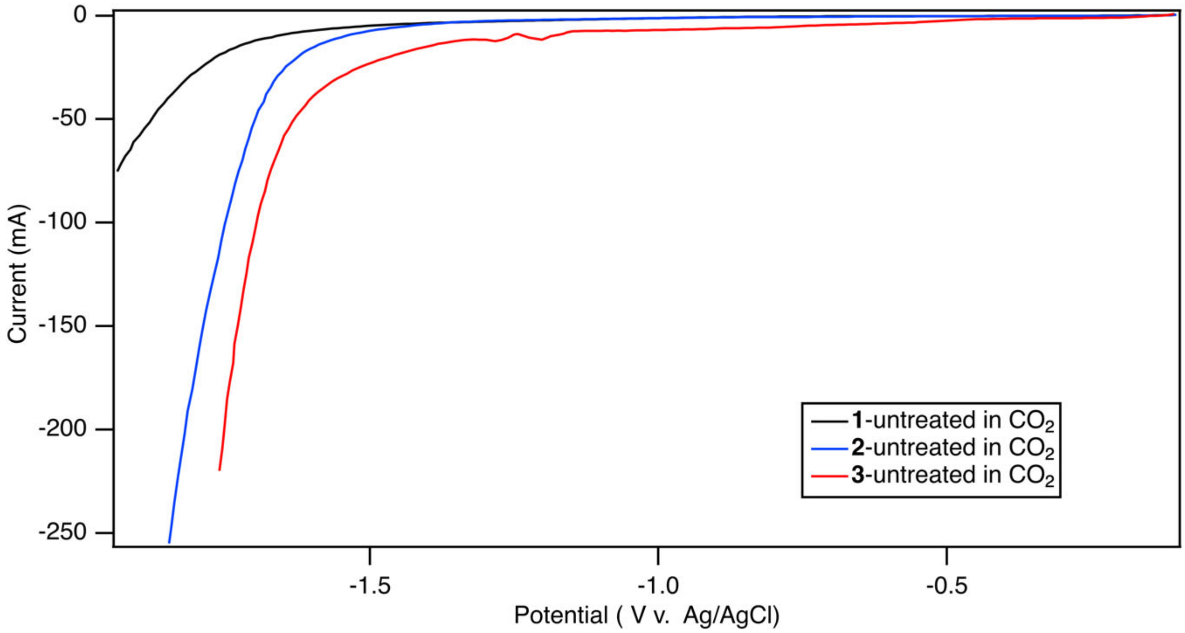

Figure S16. LSV of **1,2,3-**untreated under CO_2.

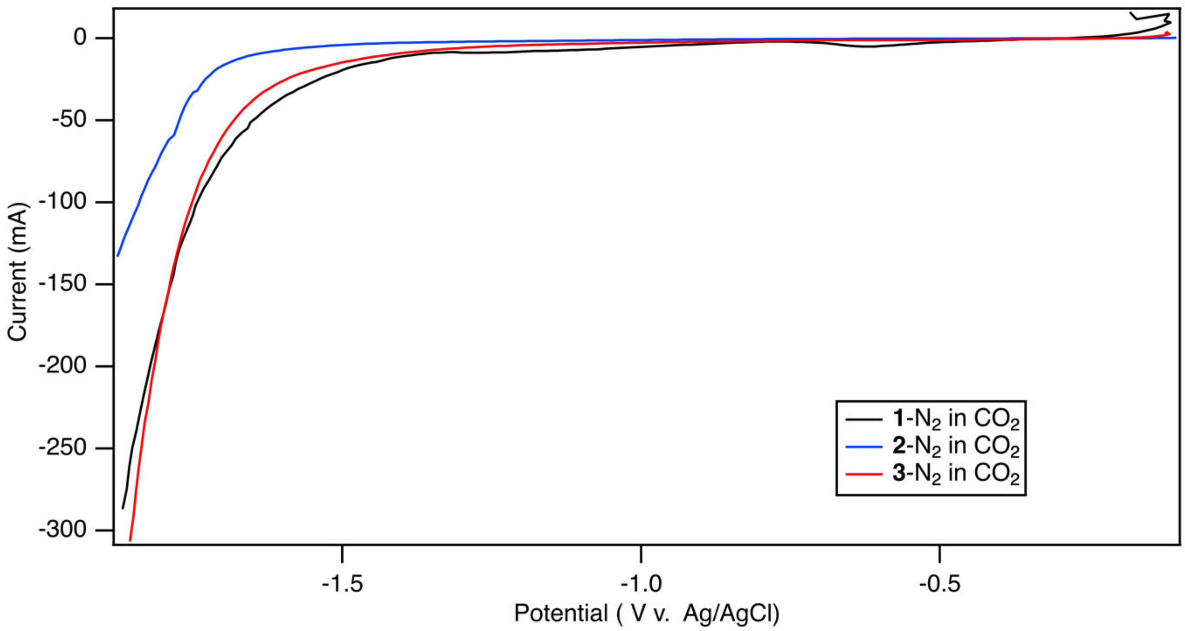

Figure S17. LSV of **1,2,3-**N_2 under CO_2.

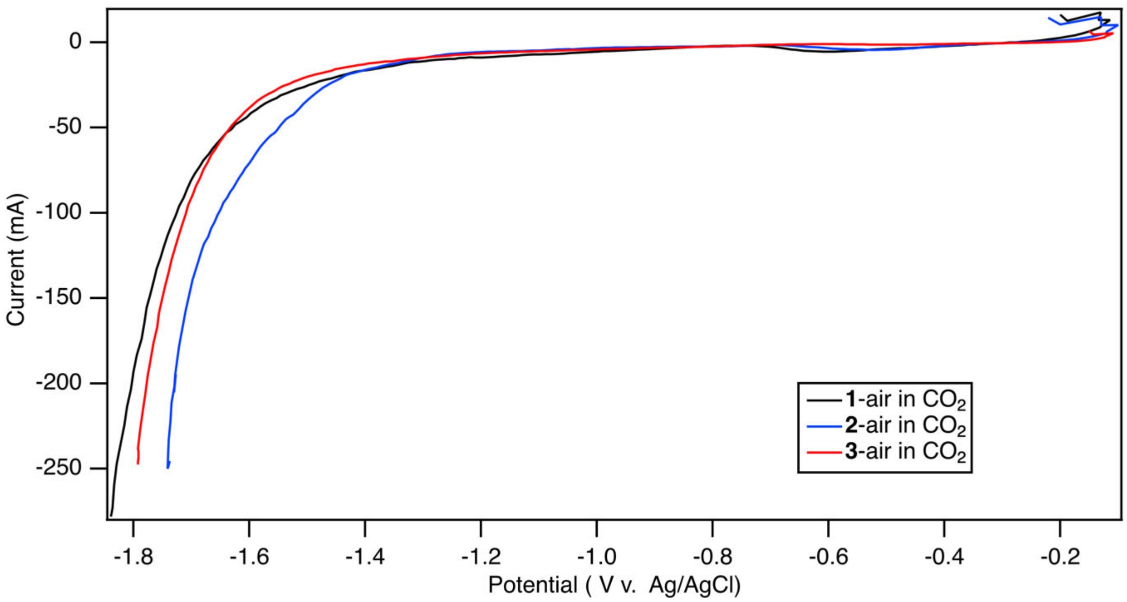

Figure S18. LSV of **1,2,3-**air under CO_2.

3. Electrochemical Catalysis.

3.1. Gas Phase Product Distribution

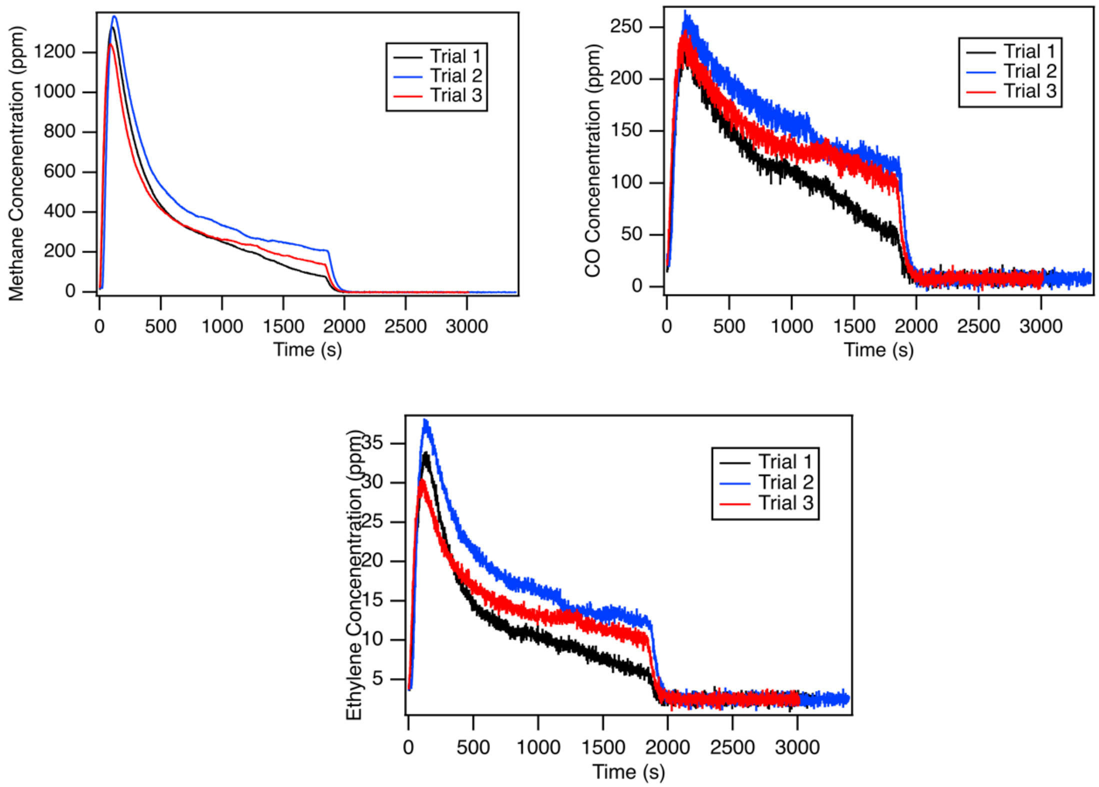

Figure S19. Observed gas concentrations for **1**-untreated across three trials.

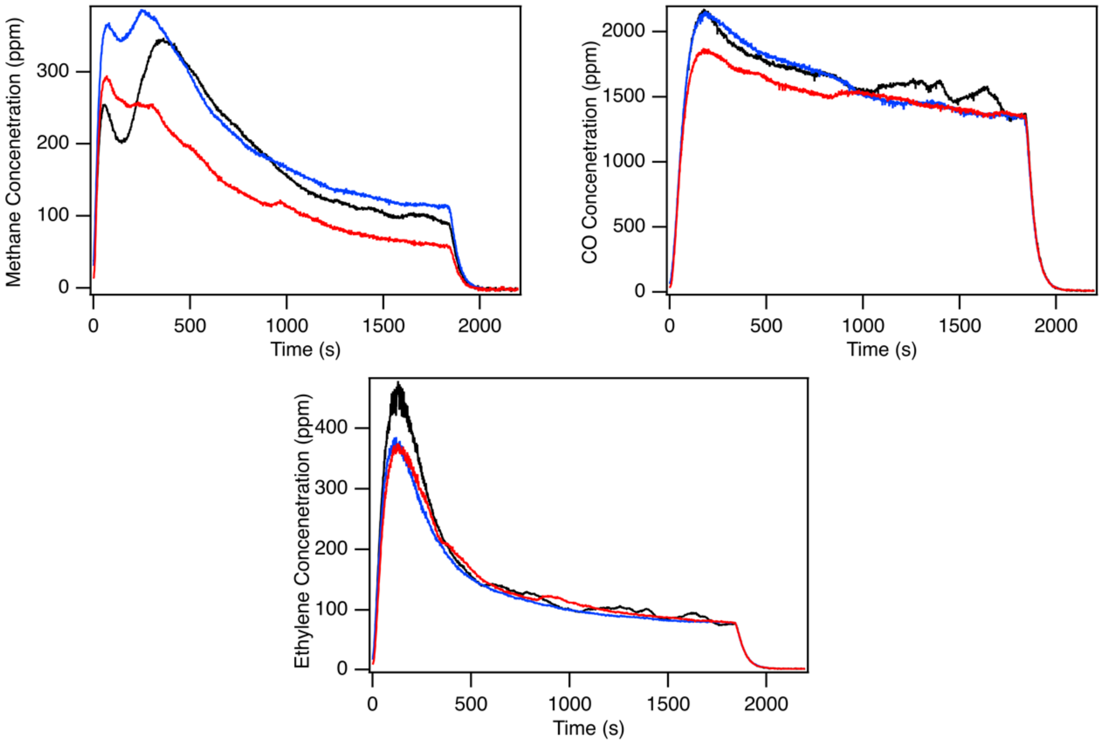

Figure S20. Observed gas concentrations for **2**-untreated across three trials.

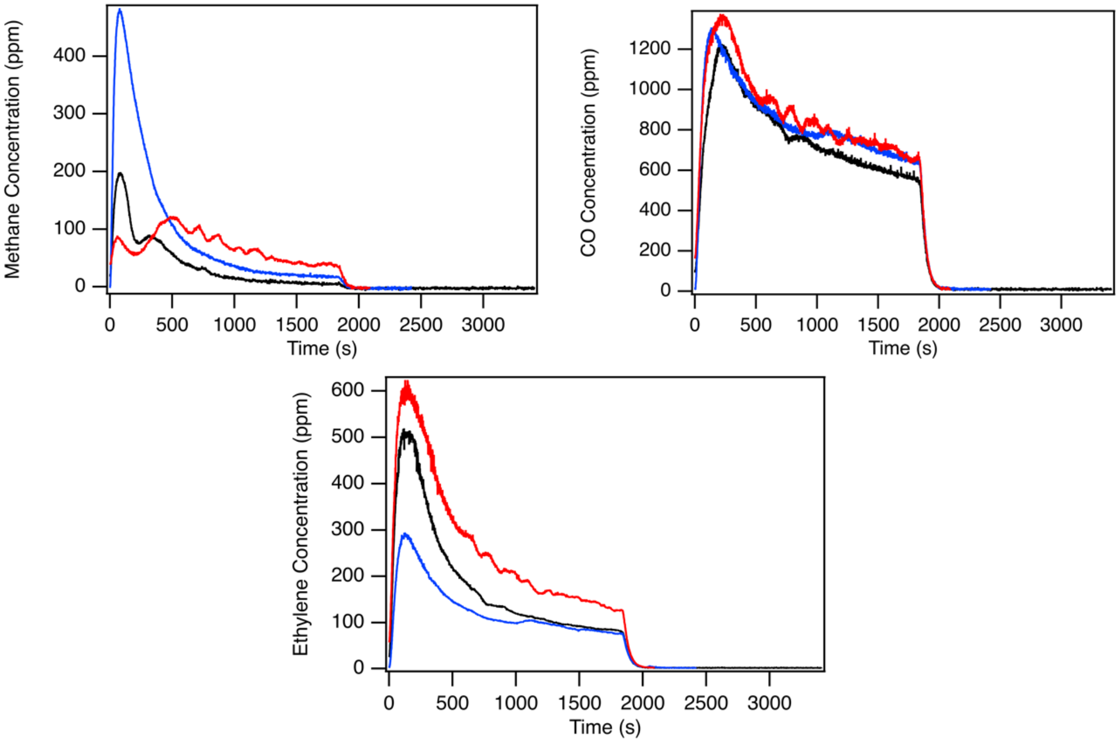

Figure S21. Observed gas concentrations for **3**-untreated across three trials.

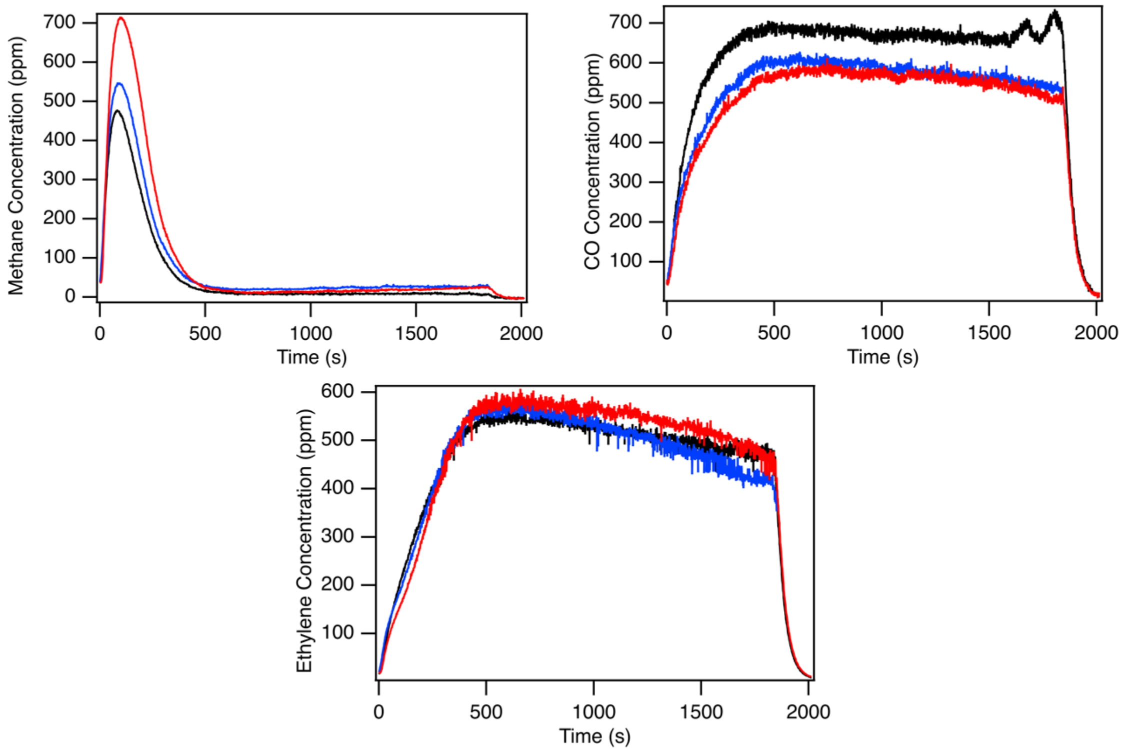

Figure S22. Observed gas concentrations for **1**-N_2 across three trials.

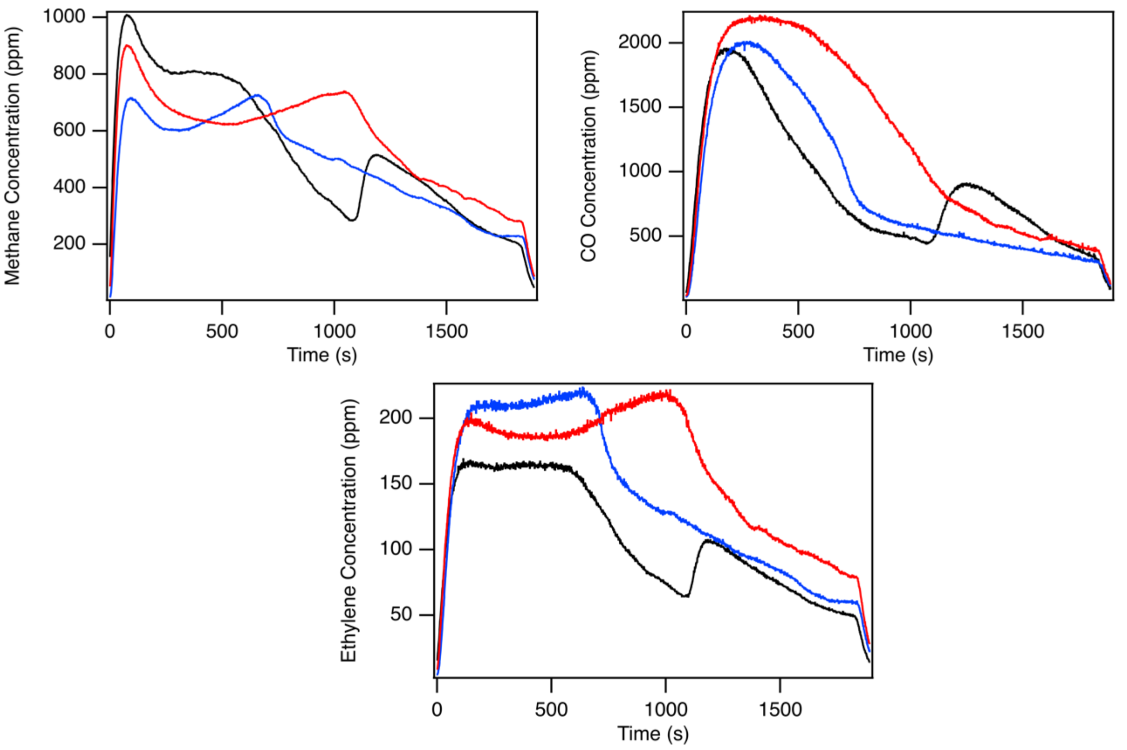

Figure S23. Observed gas concentrations for **2**-N_2 across three trials.

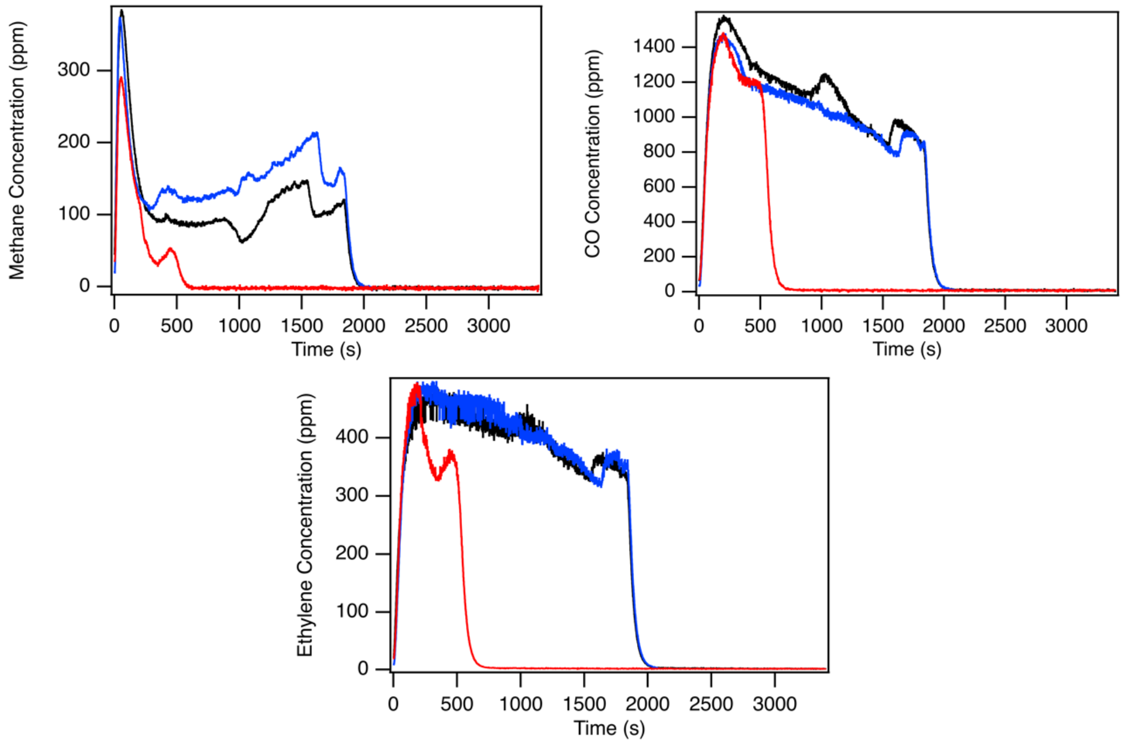

Figure S24. Observed gas concentrations for **3**-N_2 across three trials.

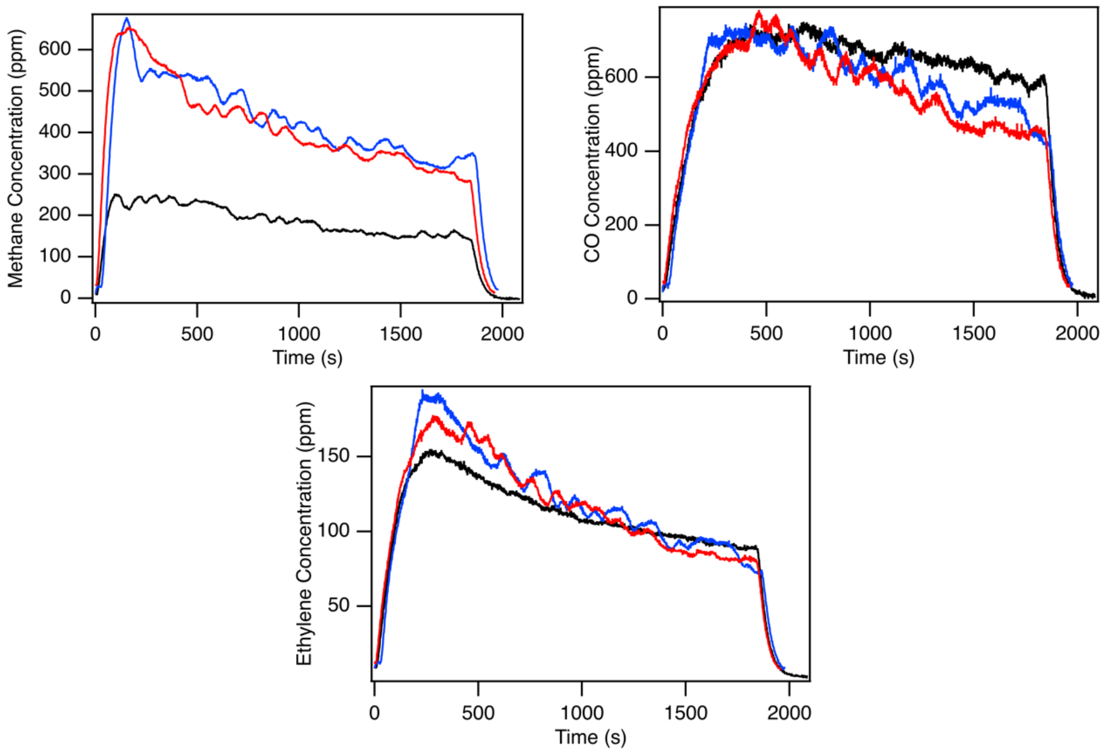

Figure S25. Observed gas concentrations for **1**-air across three trials.

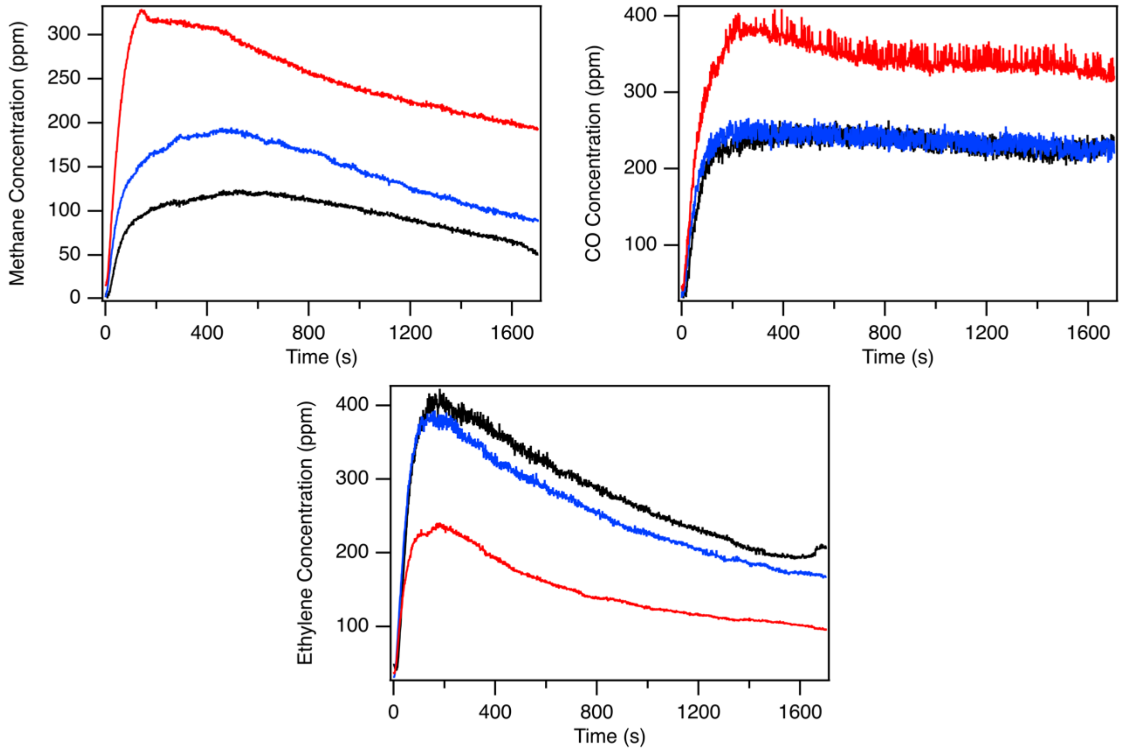

Figure S26. Observed gas concentrations for **2**-air across three trials.

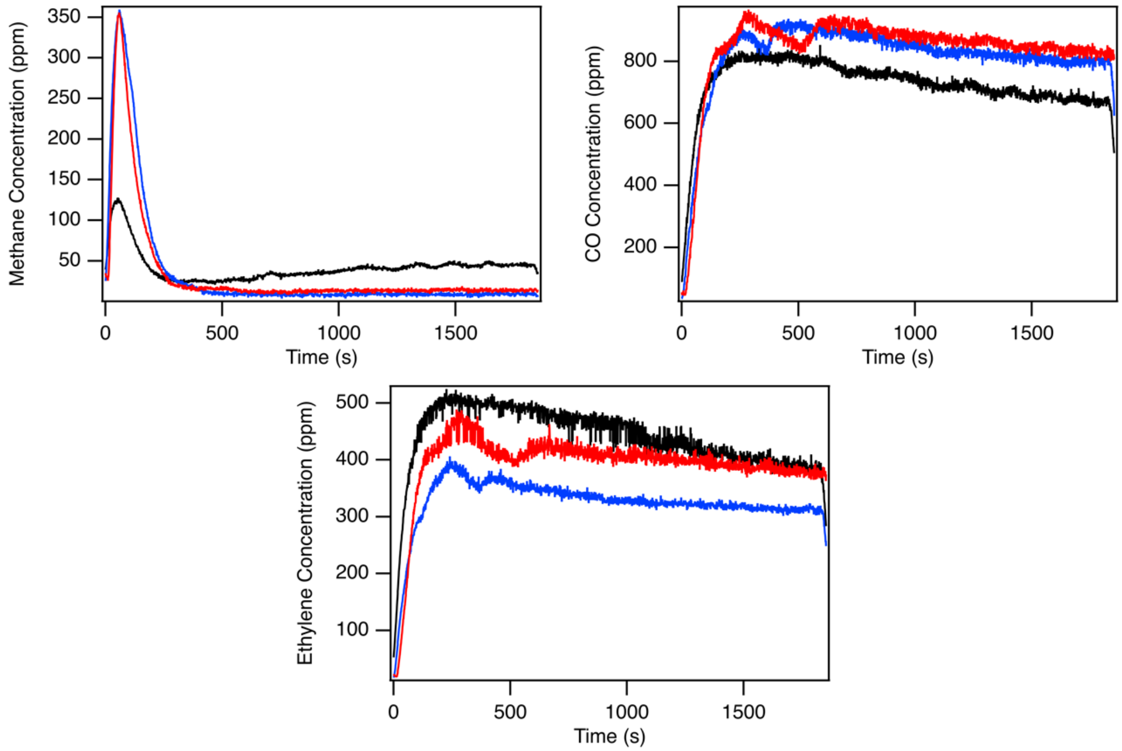

Figure S27. Observed gas concentrations for **3**-air across three trials.

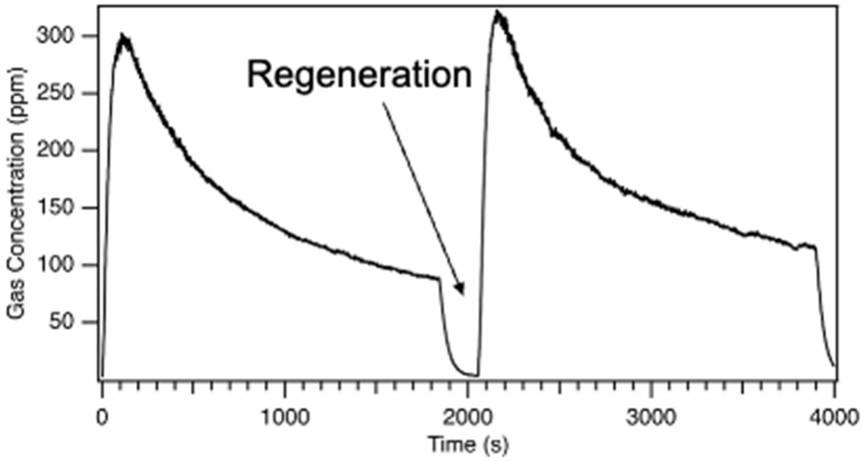

Figure S28. Regeneration of catalyst, monitoring product distribution over time.

4. Product Analysis.

4.1. Analysis of Faradaic Efficiencies for Gaseous Products. The Faradaic Efficiency calculations of the gas products as previously reported.[1] Faradaic Efficiency was recorded during the sum of reaction over the course of 1800 s and followed equation S1. There, FE_g is the gas faradaic efficiency, n_e is the number of electrons requires, x is the ppm of gas formed, P = 101325 Pa, T = 298 K, F is Faraday's Constant, R is the gas constant, and I is the average current density.

$$FE_g = \frac{n_e(x)(F)(flow\ rate)(P)}{RTI} \qquad (S1)$$

4.2. Analysis of Liquid Products via NMR Quantitation. ^{1}H NMR was recorded on a Bruker Avance 400 at room temperature. Chemical shifts were recorded relative to solvent resonances.[2] For quantitation, dimethylsulfone was used as an internal standard following Eq. S2. There C_x is the unknown concentration, N is the number of nuclei involved in the signal (i.e., N = 6 for dimethylsulfone, N = 1 for formate, N = 3 for acetate, N = 3 for ethanol), and C_{cal} is the concentration of the calibrant.

$$C_x = \frac{I_x}{I_{cal}} \times \frac{N_{cal}}{N_x} \times C_{cal} \qquad (S2)$$

The calibrant stock solution was made by diluting a mixture of dimethylsulfone (0.0757 g, 0.804 mmol) in D_2O (10.625 mL). Solutions for measurement were prepared by adding anolyte/catholyte solution (0.50 mL) with calibrant stock (0.020 mL). This gave rise to the ^1H NMR spectra recorded in Figure S29-S37. Relative integrations were recorded to dimethylsulfone (3.10 – 2.92 ppm). Integral regions for formate (8.35 – 8.25 ppm), acetate (1.85-1.65), and ethanol (1.065-0.972) were defined and used for all samples. Integrations recorded are shown in Table S2. Concentrations derived from the integral values, corrected for standard addition, are shown in Table S2 and added concentrations are shown in Table S3.

4.3. Faradaic Efficiency Calculations of Liquid Products. Faradaic Efficiency calculations of liquid products were performed previously reported.[1] In summary, total quantities from integrated NMR measurements were used in Eq S3. There N is the number of moles of liquid product formed and n is the number of electrons required to transfer. Q_{total} is the total amount of charge transferred.

$$FE_l = \frac{NnF}{Q_{total}} \qquad (S3)$$

Table S2. ^{1}H NMR Integrations relative to dimethylsulfoxide internal standard.

	Formate	Acetate	Ethanol
**1**-untreated (catholyte)	0.0381	0.0967	tr
**1**-untreated (anolyte)	0.0092	0.0818	tr
**2**-untreated (catholyte)	0.218	0.0671	0.0540
**2**-untreated (anolyte)	0.0168	0.0681	tr
**3**-untreated (catholyte)	0.3085	0.1055	0.0638
**3**-untreated (anolyte)	0.0203	0.04037	tr
**1**-N_2 (catholyte)	0.1616	0.0972	0.2379
**1**- N_2 (anolyte)	0.0044	0.113	tr
**2**- N_2 (catholyte)	0.0637	0.0798	0.0506
**2**- N_2 (anolyte)	0.0052	0.0895	tr
**3**- N_2 (catholyte)	0.2203	0.1646	0.1578
**3**- N_2 (anolyte)	0.0299	0.2043	tr
**1**-air (catholyte)	0.0616	0.0930	0.0207
**1**- air (anolyte)	0.0730	0.1825	0.0495
**2**- air (catholyte)	0.2837	0.0629	0.2068
**2**- air (anolyte)	0.0273	0.2705	tr
**3**- air (catholyte)	0.1065	0.1982	0.1584
**3**- air (anolyte)	tr	0.1085	tr

tr = trace

Table S3. Standardized Concentrations (mM) Calculated from NMR Integrations utilizing Eq S2.

	Formate	Acetate	Ethanol
**1**-untreated (catholyte)	0.69	0.59	tr
**1**-untreated (anolyte)	0.17	0.50	tr
**2**-untreated (catholyte)	3.96	0.41	0.32
**2**-untreated (anolyte)	0.31	0.41	tr
**3**-untreated (catholyte)	5.60	0.64	0.39
**3**-untreated (anolyte)	0.37	0.24	tr
**1**-N_2 (catholyte)	2.94	0.59	1.44
**1**- N_2 (anolyte)	0.09	0.68	tr
**2**- N_2 (catholyte)	1.15	0.48	0.31
**2**- N_2 (anolyte)	0.09	0.54	tr
**3**- N_2 (catholyte)	4.00	1.00	0.96
**3**- N_2 (anolyte)	0.54	1.23	tr
**1**-air (catholyte)	1.11	0.56	0.13
**1**- air (anolyte)	1.32	1.10	0.30
**2**- air (catholyte)	5.15	0.38	1.25
**2**- air (anolyte)	0.49	1.63	tr
**3**- air (catholyte)	1.93	1.20	0.96
**3**- air (anolyte)	tr	0.66	tr

tr = trace

Table S4. Total Produced Quantity of Liquid Products (mmol) Produced in the Flow Cell Reactions.

	Formate	Acetate	Ethanol
**1**-untreated	0.017	0.021	tr
**2**-untreated	0.085	0.016	0.006
**3**-untreated	0.119	0.018	0.008
**1**-N_2	0.060	0.025	0.029
**2**-N_2	0.025	0.021	0.006
**3**-N_2	0.091	0.045	0.019
**1**-air	0.049	0.033	0.008
**2**-air	0.113	0.040	0.025
**3**-air	0.039	0.037	0.019

tr = trace

Table S5. Faradaic Efficiencies for **1-3.**

Catalyst	Ethylene	Methane	CO	Formate	Acetate	Ethanol
**1**-untreated	0.99	17.25	1.51	0.73	3.7	ND
**1**-air	7.37	14.5	6.22	2.1	5.72	2.18
**1**-N_2	30.09	3.4	5.98	2.59	4.37	7.41
**2**-untreated	9.34	7.57	16.54	3.65	2.81	1.68
**2**-air	13.78	6.68	2.74	4.84	6.92	6.44
**2**-N_2	8.72	22.93	10.86	1.07	3.51	1.57
**3**-untreated	12.28	2.96	8.74	5.12	3.03	1.98
**3**-N_2	24.41	1.55	8.37	1.65	6.37	4.93
**3**-air	25.24	5.76	11.46	3.89	7.66	4.91

ND = not detected

Table S6. Peak Faradaic Efficiencies for Gaseous Products.

	Peak CO FE	Peak CH_4 FE	Peak C_2H_4 FE
**1-Untreated**	2.65 ± 0.14	55.56 ± 2.98	2.16 ± 0.24
**1-Air**	6.92 ± 0.70	24.44 ± 5.14	37.15 ± 1.28
**1-N_2**	10.17 ± 3.79	19.20 ± 8.38	12.76 ± 2.56
**2-Untreated**	21.95 ± 1.50	15.10 ± 2.08	27.75 ± 4.50
**2-Air**	21.73 ± 1.42	37.02 ± 5.99	12.95 ± 1.95
**2-N_2**	3.77 ± 1.78	6.19 ± 2.58	23.91 ± 2.89
**3-Untreated**	16.09 ± 3.64	5.69 ± 2.31	37.12 ± 3.47
**3-Air**	16.29 ± 3.44	12.26 ± 5.06	29.83 ± 3.15
**3-N_2**	9.66 ± 0.62	11.82 ± 5.58	29.85 ± 3.84

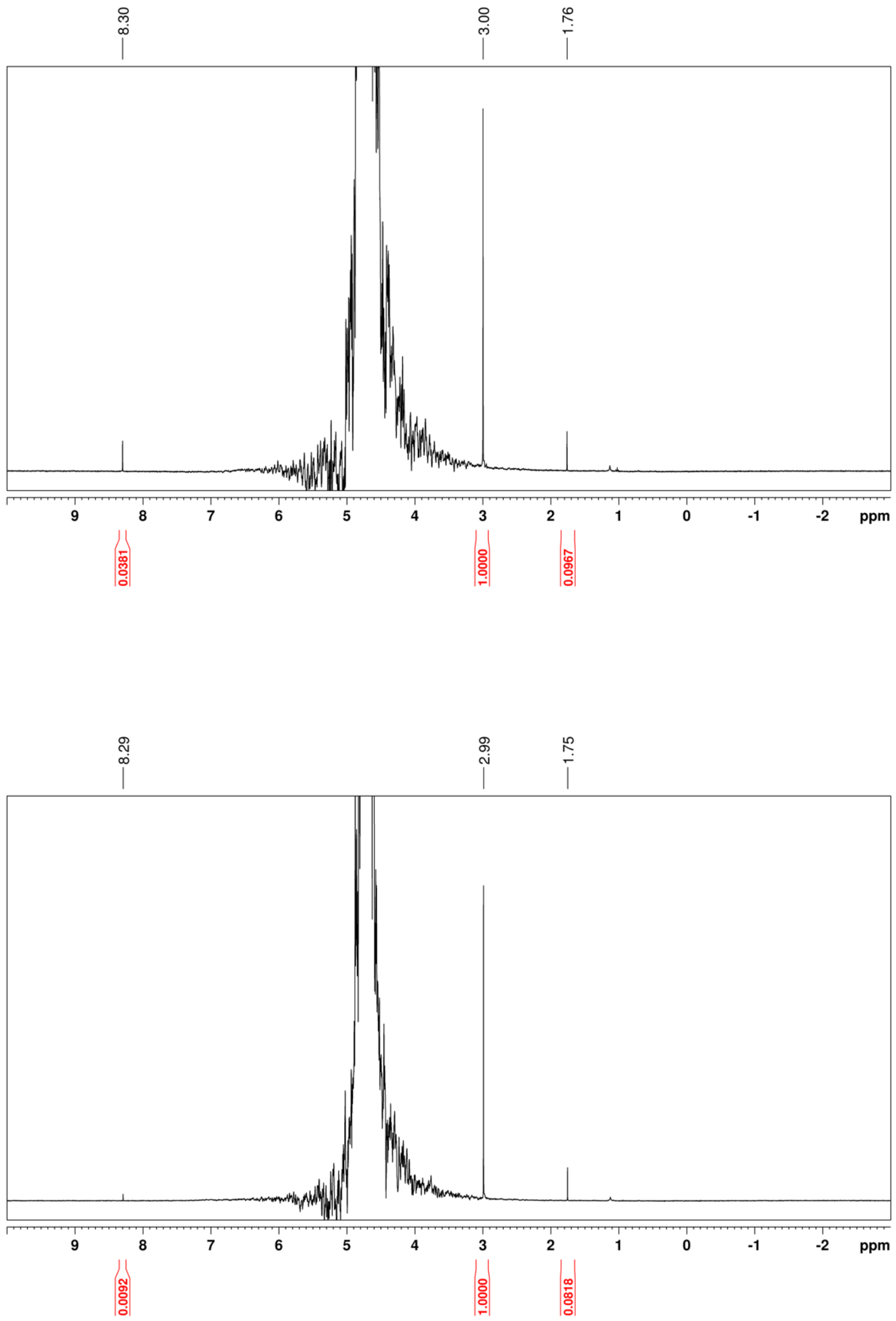

Figure S29. ^{1}H NMR of **1**-untreated of the (Top) catholyte and (Bottom) anolyte from 10.00 ppm to –3.00 ppm recorded in H_2O solution with D_2O lock.

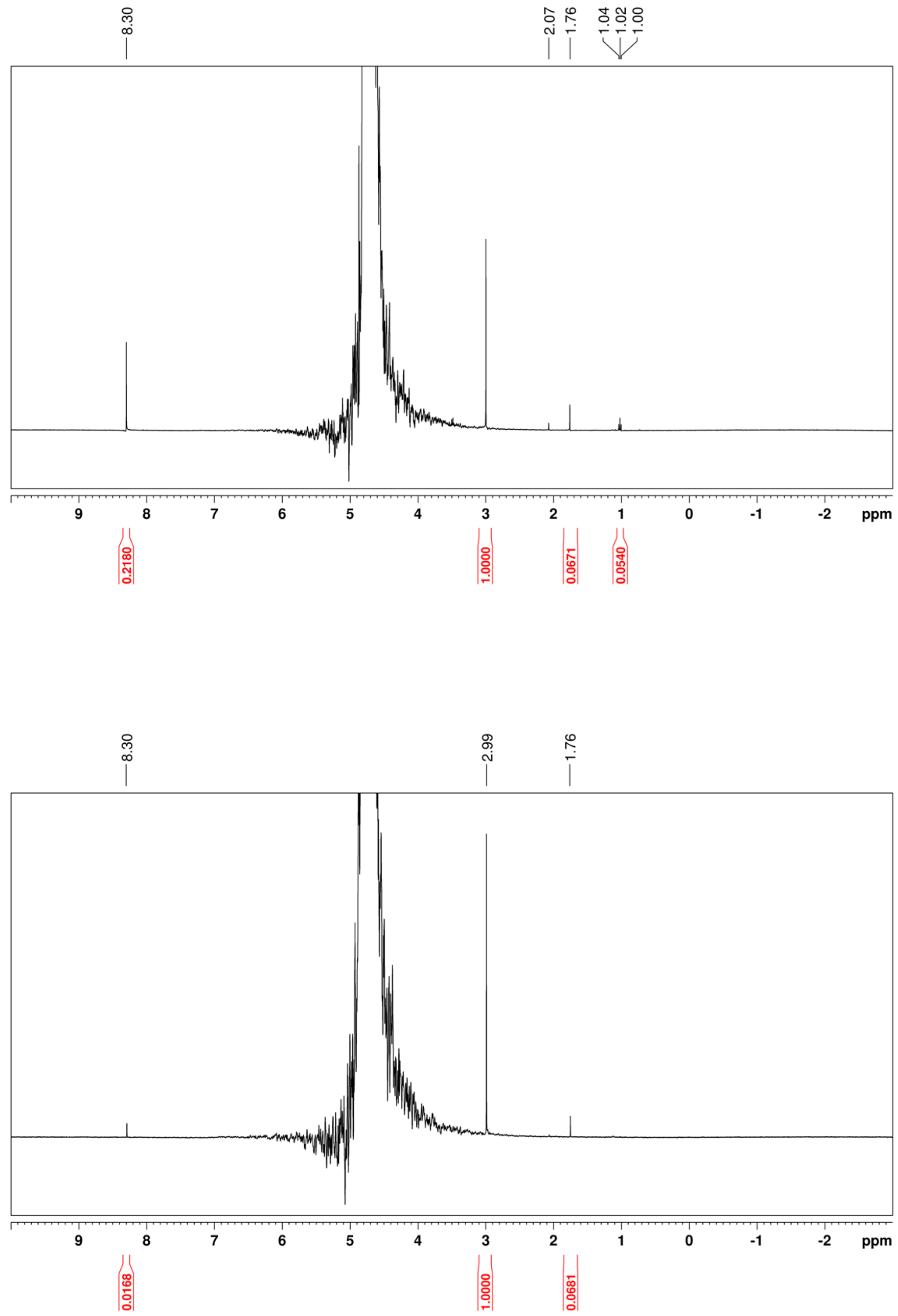

Figure S30. ^{1}H NMR of **2**-untreated of the (Top) catholyte and (Botttom) anolyte from 10.00 ppm to –3.00 ppm recorded in H_2O solution with D_2O lock.

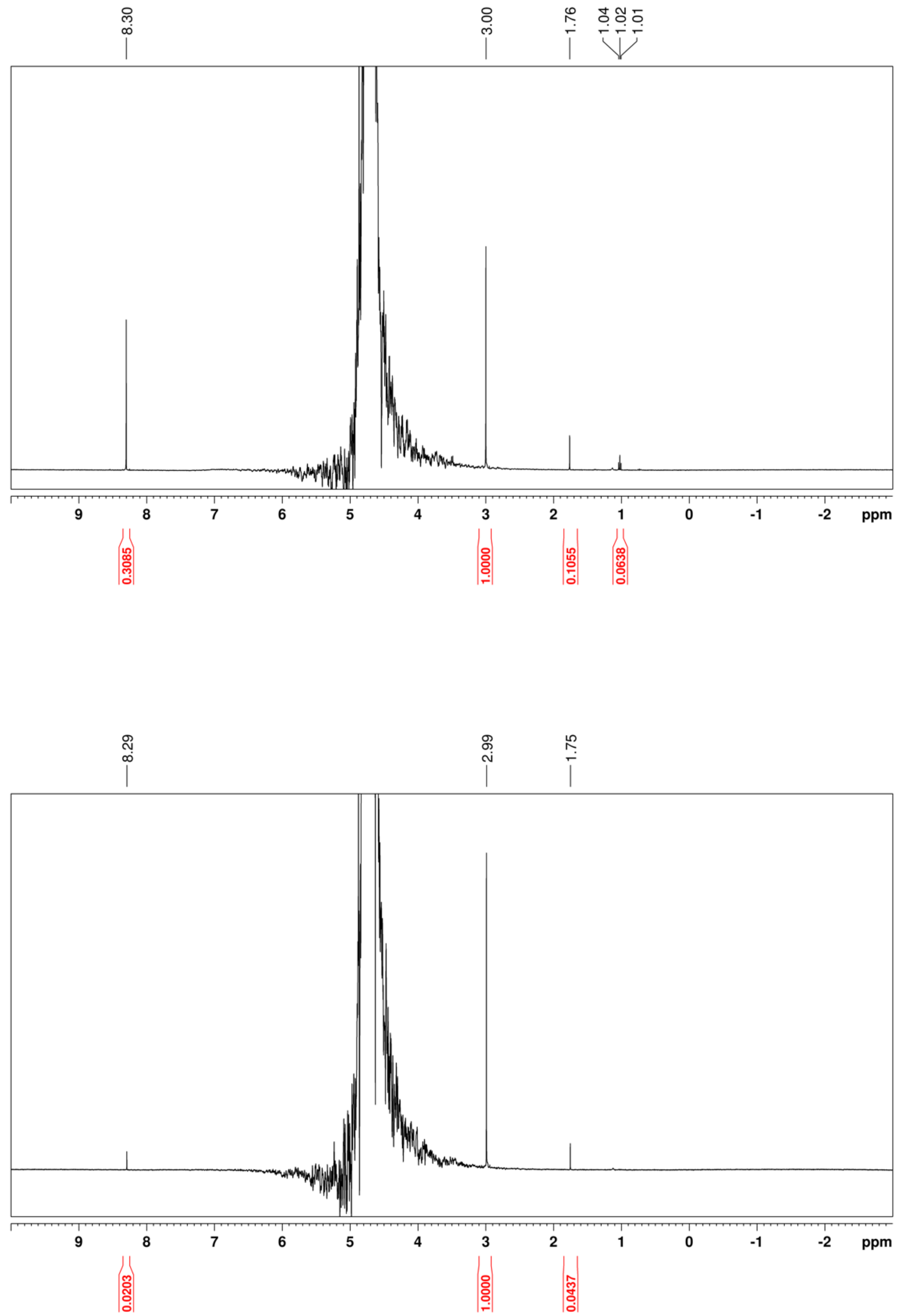

Figure S31. ^1H NMR of **3**-untreated of the (Top) catholyte and (Bottom) anolyte from 10.00 ppm to –3.00 ppm recorded in H_2O solution with D_2O lock.

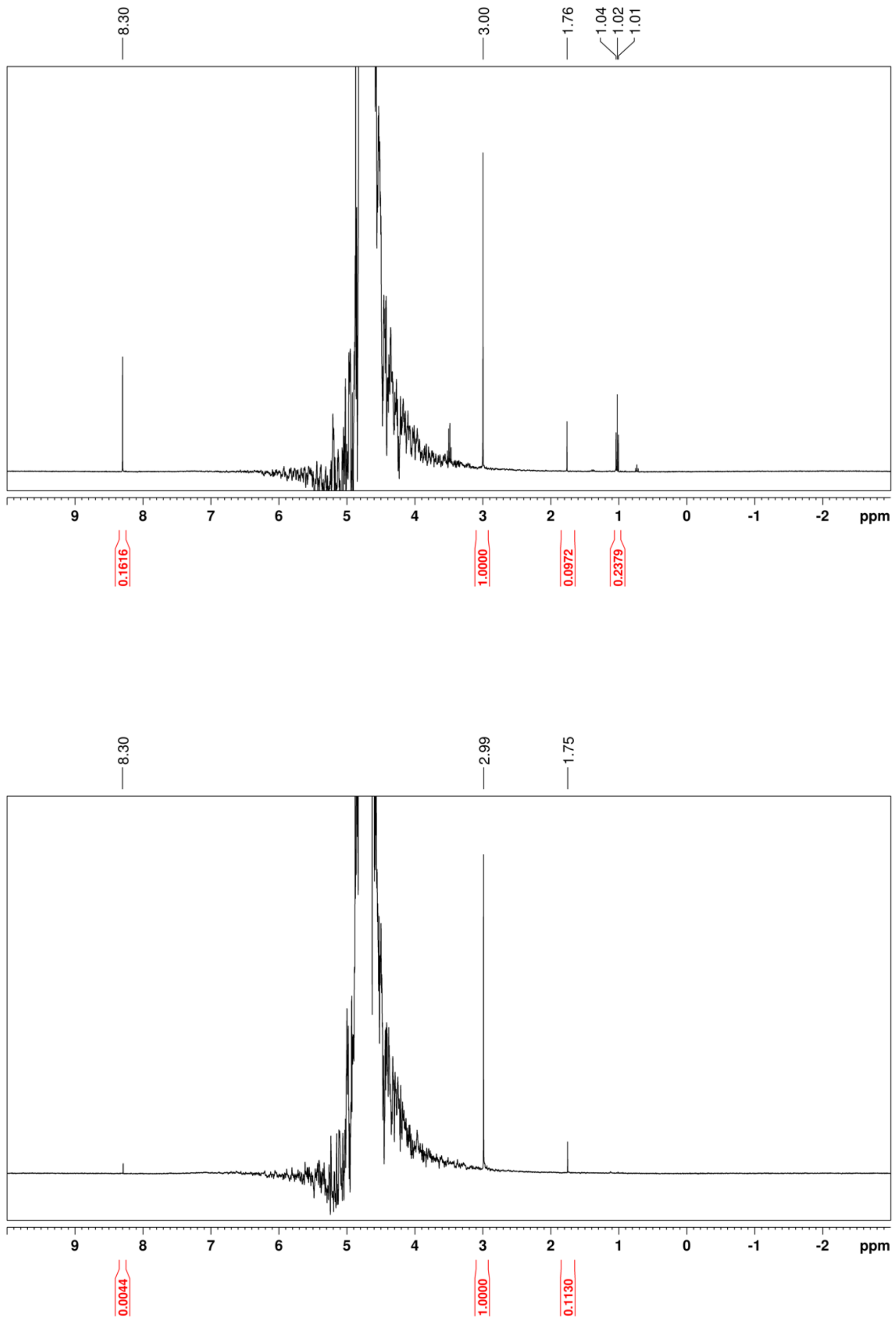

Figure S32. ^{1}H NMR of **1**-N_2 of the (Top) catholyte and (Bottom) anolyte from 10.00 ppm to –3.00 ppm recorded in H_2O solution with D_2O lock.

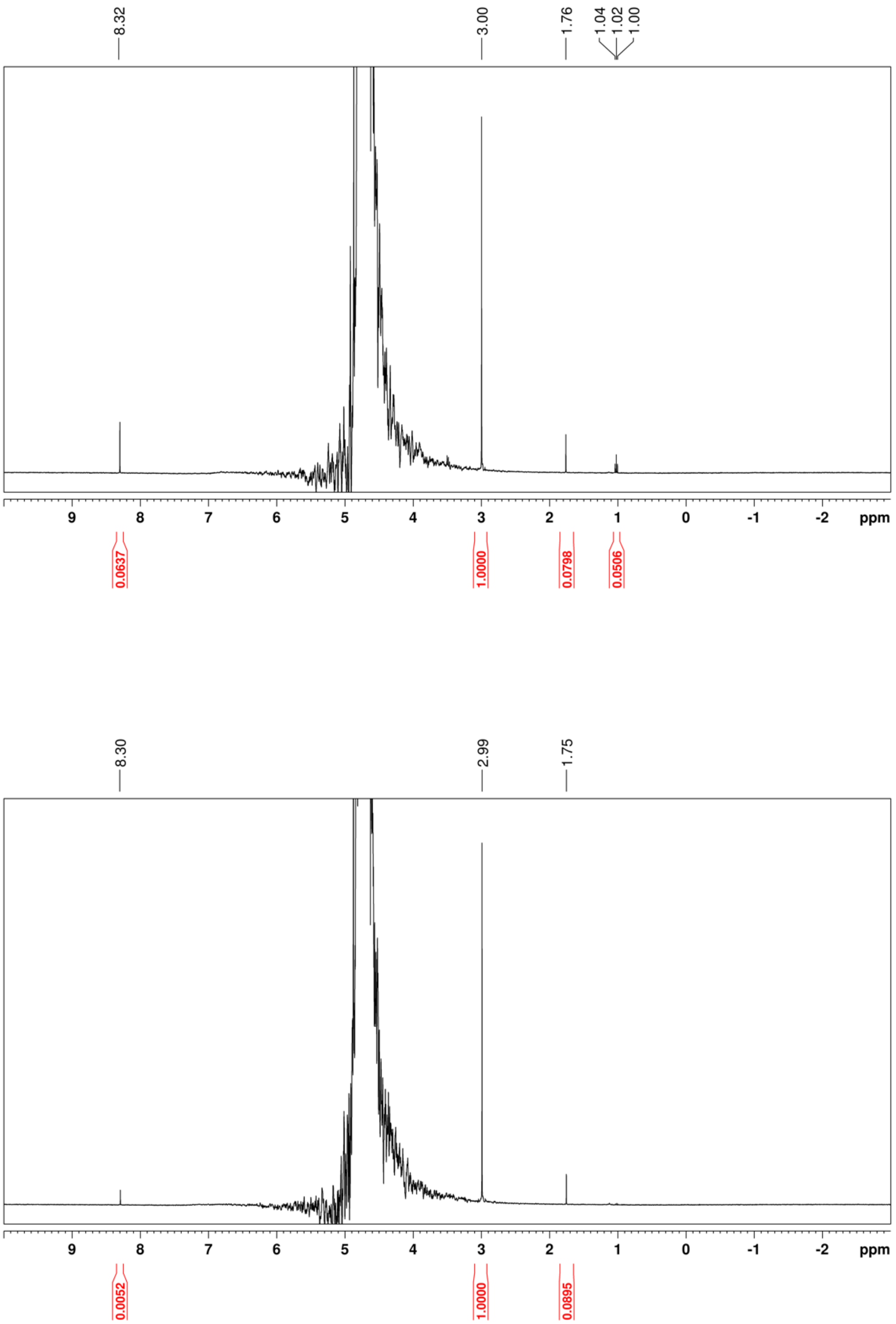

Figure S33. ^{1}H NMR of **2**-N_2 of the (Top) catholyte and (Bottom) anolyte from 10.00 ppm to –3.00 ppm recorded in H_2O solution with D_2O lock.

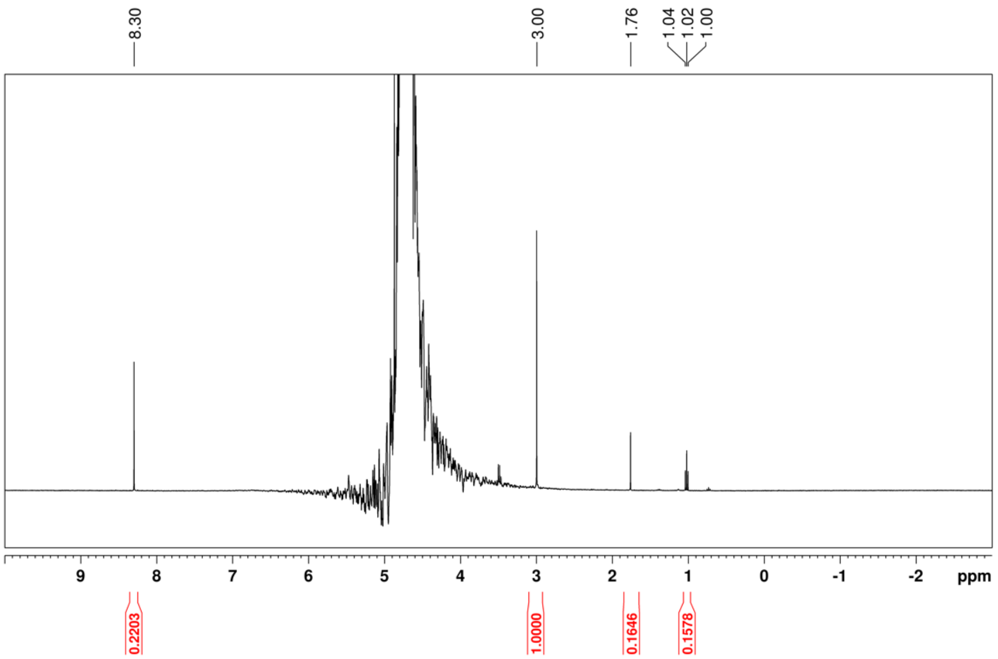

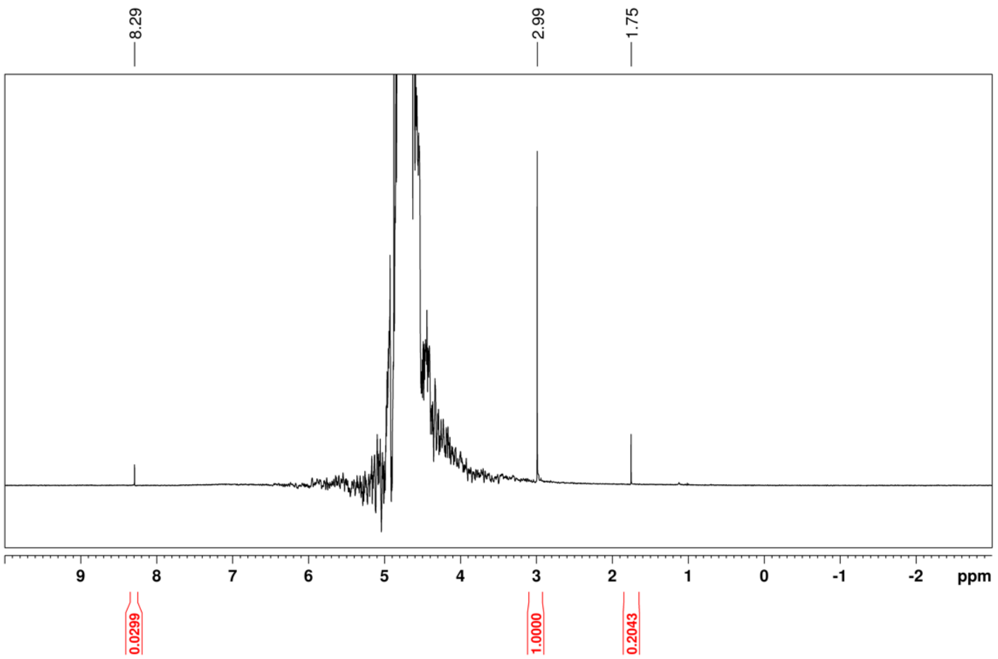

Figure S34. ^{1}H NMR of **3**-N_2 of the (Top) catholyte and (Bottom) anolyte from 10.00 ppm to –3.00 ppm recorded in H_2O solution with D_2O lock.

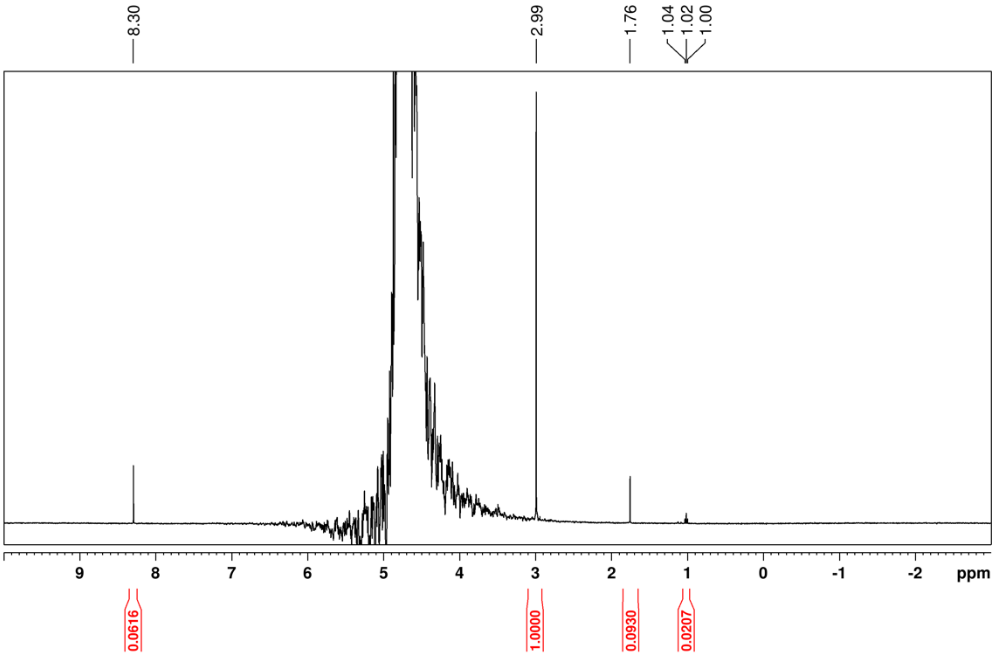

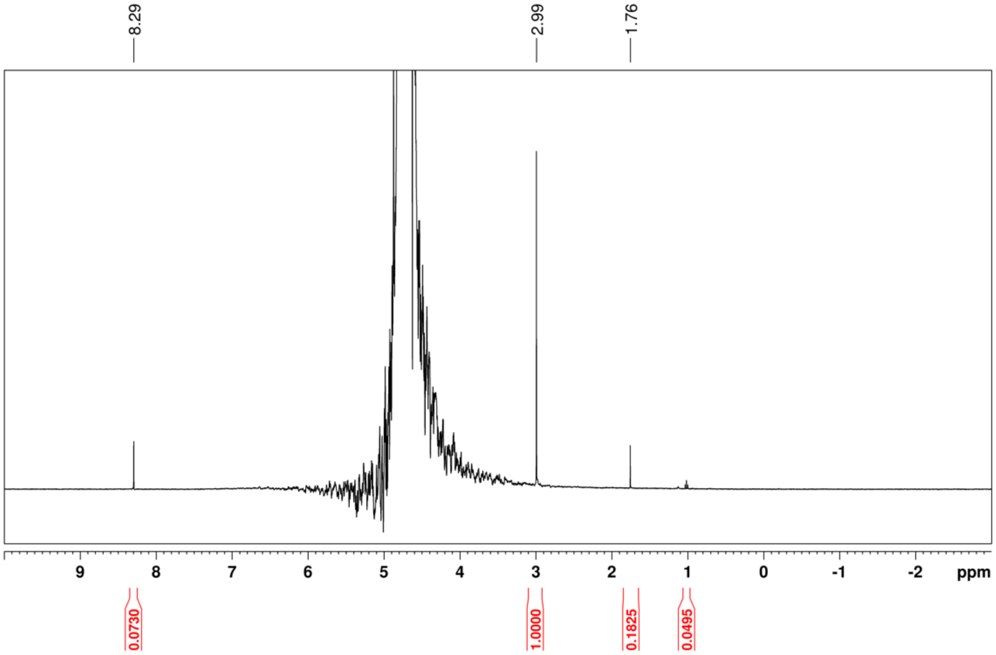

Figure S35. ^{1}H NMR of **1**-air of the (Top) catholyte and (Bottom) anolyte from 10.00 ppm to – 3.00 ppm recorded in H_2O solution with D_2O lock.

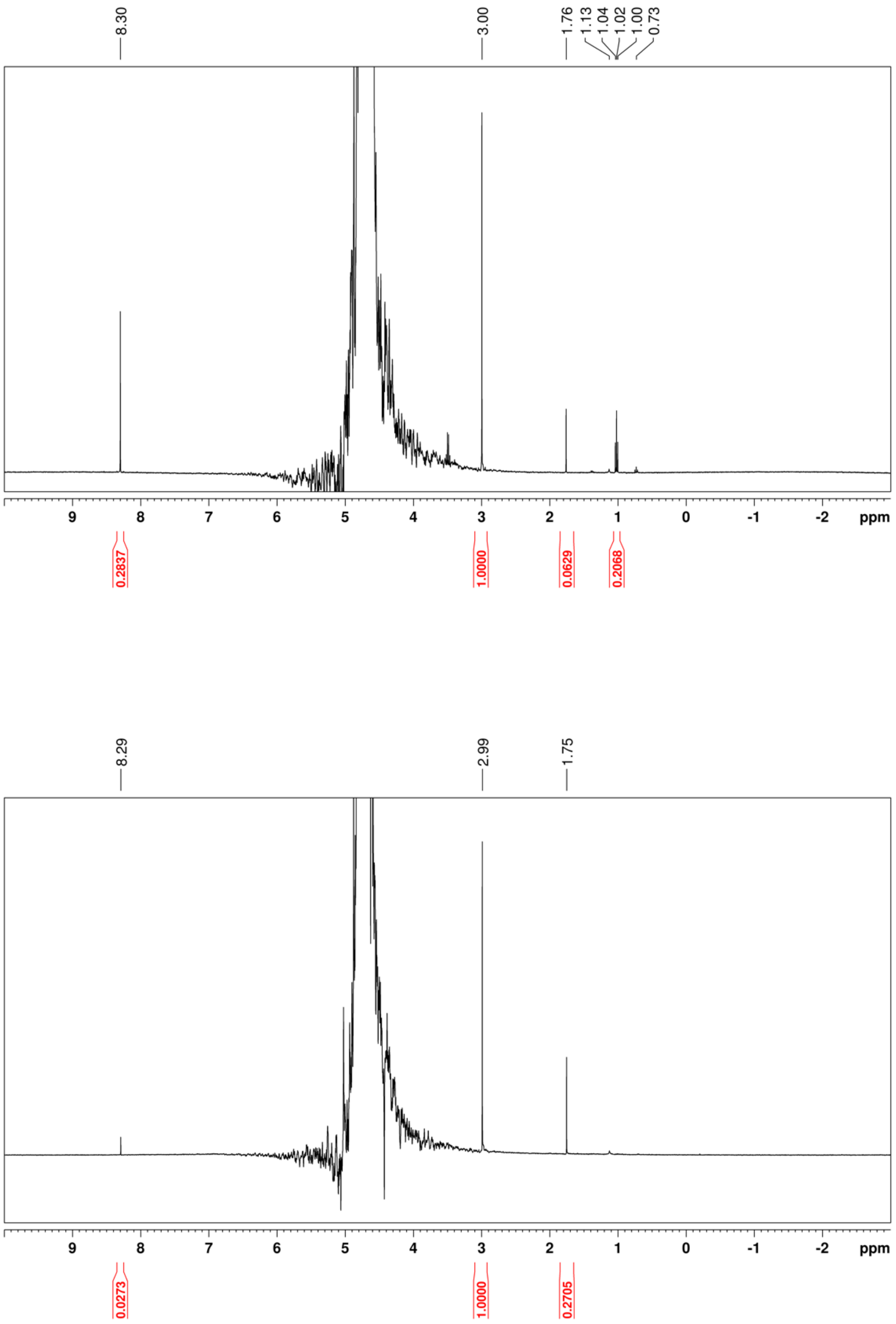

Figure S36. ^{1}H NMR of **2**-air of the (Top) catholyte and (Bottom) anolyte from 10.00 ppm to –3.00 ppm recorded in H_2O solution with D_2O lock.

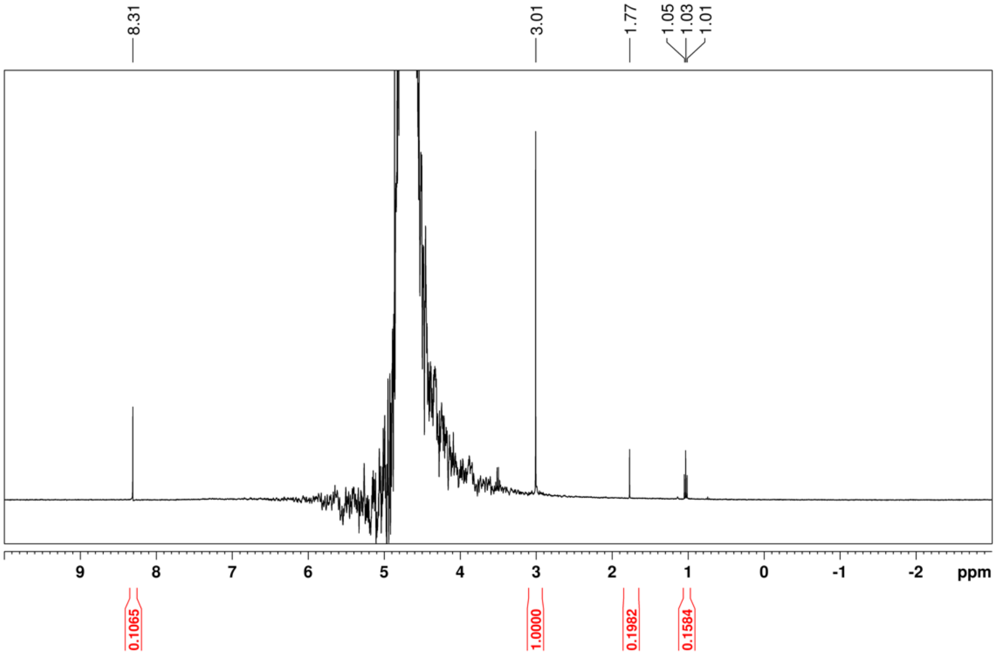

Figure S37. ^{1}H NMR of **1**-air of the (Top) catholyte and (Bottom) anolyte from 10.00 ppm to –3.00 ppm recorded in H_2O solution with D_2O lock.

4.4 Cost Analysis.

Estimated Material Costs per kg

$CuCO_3$: 123.55 g/mol
Cu + C product (assuming 1/1 stoichiometry) = 73.55 g/mol
Yield: 59.5%

1 kg of Cu + C requires 1.68 kg of $CuCO_3$
Assume Copper Carbonate spot price of $5/kg
Cost of 1 kg of Cu + C catalyst = $5/kg $CuCO_3$ * 1.68kg CuCO3/1kg Cu+C = $8.4/kg catalyst

Energy Cost per kg

Maximum furnace power draw = 2 kW
Synthesis Duration = 4 hours
2 kW*(4h) = 8 kWh
Avg energy cost in USA = $0.0795 /kWh
Total Cost = $0.636
Total Cost for 1 kg cat. (Materials + Energy) = $8.4 + $0.636 = $9.04/kg

References.

1 Dutta, N., Bagchi, D., Chawla, G. & Peter, S. C. A Guideline to Determine Faradaic Efficiency in Electrochemical CO_2 Reduction. *ACS Energy Lett.* **9**, 323-328 (2024). https://doi.org/10.1021/acsenergylett.3c02362

2 Fulmer, G. R. *et al.* NMR Chemical Shifts of Trace Impurities: Common Laboratory Solvents, Organics, and Gases in Deuterated Solvents Relevant to the Organometallic Chemist. *Organometallics* **29**, 2176-2179 (2010). https://doi.org/10.1021/om100106e
